# Long-term microbiome and clinical effects of a microbiome-guided personalized diet versus low-FODMAP diet in irritable bowel syndrome: A 12-month follow-up randomized controlled trial

**DOI:** 10.1080/19490976.2026.2719125

**Published:** 2026-08-20

**Authors:** Varol Tunali, Naciye Çiğdem Arslan, Gözde Derviş Haki̇m, Aycan Gündoğdu, Beyza Hilal Ermi̇ş, Mehmet Hora, Özkan Ufuk Nalbantoğlu

**Affiliations:** a Department of Microbiology, Faculty of Medicine, Izmir University of Economics, Izmir, Turkey; b Department of Medical and Surgical Sciences, UOC Gastroenterologia, Fondazione Policlinico Universitario A. Gemelli IRCCS, Rome, Italy; c Department of General Surgery, Istanbul Health and Technology University, Istanbul, Turkey; d Department of Gastroenterology, Tepecik Education and Research Hospital, Izmir, Turkey; e Department of Immunology, Faculty of Medicine, Medeniyet University, İstanbul, Turkey; f Faculty of Nutrition and Dietetics, Adnan Menderes University, Aydın, Turkey; g Bioinformatics Division, Genome and Stem Cell Center, Erciyes University, Kayseri, Turkey; h Department of Computer Engineering, Faculty of Engineering, Dokuz Eylül University, Izmir, Turkey

**Keywords:** Irritable bowel syndrome, microbiome, low-FODMAP diet, personalized diet, precision nutrition, artificial intelligence, 16S rRNA sequencing, alpha diversity, dietary intervention, randomized trial

## Abstract

Dietary therapy is central to irritable bowel syndrome (IBS) management, yet the long-term durability of the low-FODMAP diet (LFD), and of microbiome-guided personalization, remains unclear. We assessed the long-term clinical and gut-microbiome effects of a microbiome-guided personalized diet (PD) compared with a standard LFD in adults meeting Rome IV criteria for IBS. In this multicenter, open-label randomized controlled trial with blinded outcome assessment, participants who completed a 6-week dietary intervention (PD or LFD) were followed at 6 and 12 months without further dietary intervention. Outcomes included the IBS Severity Scoring System (IBS-SSS), IBS Quality of Life (IBS-QOL), and the Hospital Anxiety and Depression Scale (HADS); gut microbiota were profiled by 16S rRNA sequencing. Longitudinal changes were evaluated using linear mixed-effects models, responder analyses, PERMANOVA, and PERMDISP. Both diets reduced IBS-SSS at 6 weeks. PD maintained symptom improvement at 6 and 12 months (−82.0 and −78.3 points from baseline), whereas LFD benefits regressed by 12 months (+29.3 points; between-group *p* = 0.001). At 12 months, IBS-SSS responder rates were higher with PD than LFD (62.5% vs 34.5%; absolute risk difference +28.0%, 95% CI 4.2–47.7; Fisher *p* = 0.029), and IBS-QOL, HADS-anxiety, and HADS-depression showed more favourable trajectories with PD. PD was associated with sustained Shannon alpha-diversity gains (+0.488 at 6 weeks; +0.205 at 12 months; both *p* < 0.01). A modest between-group beta-diversity difference at 6 months (R^2^ = 0.035; *p* = 0.011) was not significant at 12 months. This hypothesis-generating follow-up suggests more durable benefit with PD; larger trials powered for long-term clinical and microbiome outcomes are warranted.

## Graphical abstract

**Figure:**
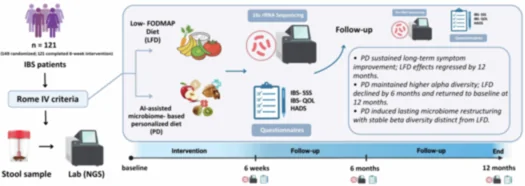

## 1. Introduction

Irritable bowel syndrome (IBS) is a complex and prevalent functional gastrointestinal disorder characterized by recurrent abdominal pain, altered bowel habits, and bloating.^1^ With a global prevalence of approximately 4.1%, IBS not only poses a serious threat to public health by impairing quality of life (QOL) but also results in substantial economic burdens through healthcare expenses and productivity losses.^2^ ^,^ ^3^ The multifactorial pathophysiology of IBS involves dysregulation of gut–brain interactions, altered gut motility, visceral hypersensitivity, and perturbations in the gut microbiome.^4^ Recent studies have underscored the pivotal role of the gut microbiome—a diverse ecosystem of microorganisms that interact with the host immune system, modulate gut barrier function, and metabolize dietary substrates—in both the development and symptomatology of IBS.^5^ ^,^ ^6^

Dietary interventions, notably the low-fermentable oligosaccharides, disaccharides, monosaccharides, and polyols (low-FODMAP; LFD) diet, have emerged as promising strategies for IBS management by restricting fermentable carbohydrates and thereby reducing substrate availability for colonic fermentation.^7^ ^,^ ^8^ However, while short-term benefits are well established, long-term adherence to a strict LFD may adversely affect the abundance and diversity of beneficial microbial species.^7-9^ To address these concerns, a microbiome-based personalized diet (PD) has been proposed as a novel therapeutic approach. This strategy integrates microbiome profiling and artificial intelligence (AI) algorithms to tailor dietary recommendations to an individual’s unique gut microbial profile, thereby aiming not only for symptomatic relief but also for the restoration and enhancement of microbial diversity and function.^10-12^

Emerging evidence suggests that sustained modulation of the gut microbiome is crucial for long-term health. The gut microbiota plays a central role in regulating host metabolism and immune responses and has been implicated in the development of various conditions, including obesity, type 2 diabetes, and certain cancers.^13^ Understanding the molecular crosstalk between gut microbes and host systems is considered essential for advancing novel therapeutic approaches. Nutrition is recognized as a key modulator of immune function and gut microbiota composition, with significant implications for long-term health, particularly in aging populations.^14^ Emerging evidence also suggests that dietary interventions may influence epigenetic mechanisms, contributing to increased microbiome diversity and reduced systemic inflammation.^15^

Long-term IBS management necessitates interventions that offer not only short-term symptom relief but also sustained efficacy over time. In our previous work, we demonstrated that both the LFD and PD interventions can yield significant improvements in IBS symptoms.^12^ Building on these findings, the present study examines the long-term effects of a 6-week dietary intervention, assessing symptom trajectories and gut microbiome changes at 6 and 12 months post-intervention. This extended observation period allows for a comprehensive evaluation of the durability of clinical responses and the persistence of microbiota modulation.

## 2. Methods

### 2.1. Study design and participants

This study builds on a multicenter, parallel, randomized, controlled, open-label (with blinded outcome assessment; PROBE design) trial conducted between August 2022 and May 2023 across four gastroenterology outpatient clinics in Istanbul, Izmir, and Kayseri, Turkey. The original trial compared the efficacy of a PD with a standardized LFD for the management of IBS. Eligible participants were adults aged 18–65 years who fulfilled Rome IV criteria for IBS. Ethics approval was obtained from regional committees, and the study was registered at ClinicalTrials.gov (NCT05646186). Written informed consent was obtained from all participants. The trial followed CONSORT guidelines.

#### 2.1.1. Randomization and masking

Participants who met the eligibility criteria were randomly allocated to either the PD group or the LFD group using a computer-generated randomization sequence prepared by an independent researcher who was not involved in participant recruitment, intervention delivery, outcome assessment, or data analysis. The allocation sequence was placed in sequentially numbered, opaque, sealed envelopes to ensure allocation concealment. Envelopes were opened by the research dietitian only after participant eligibility had been confirmed and written informed consent had been obtained. Because dietary counseling and menu plans differed between interventions, the dietitians delivering the intervention were aware of group allocation. Participants were informed that both approaches represented evidence-based dietary strategies for IBS management but were not informed about the study hypothesis or any expected superiority of one intervention over the other. Investigators responsible for outcome assessment and data collection were not involved in the allocation process and remained unaware of treatment assignment. Statistical analyzes were performed using coded group labels so that analysts were blinded to group identity during the analysis stage.

#### 2.1.2. Microbiome-guided personalized diet generation

The PD recommendations were generated using the algorithm previously developed by Karakan et al.^10^ and applied in our parent trial;^12^ further methodological details are provided in those publications. In brief, genus-level relative abundances from each participant's baseline 16S rRNA V4 profile served as input to a supervised machine-learning classifier (gradient-boosted decision trees) that estimates an IBS-index reflecting predicted symptom burden. A Monte Carlo Metropolis search then identified an alternative microbiome configuration associated with a lower predicted IBS-index, and the required directional shifts in genus abundances were translated into per-nutrient targets and subsequently into individualized dietary recommendations. The algorithm was not retrained or modified using data from the present follow-up analysis, and no new microbiome-guided dietary optimization was performed at the 6- or 12-month follow-up visits; the present analysis therefore evaluates the durability of the baseline-generated intervention rather than repeated re-personalization. LFD arm received standardized low-FODMAP dietary advice delivered by dietitians. Counseling time, session structure, and frequency were matched between arms. Representative example plans are provided in Supplementary Files S1 (PD) and S2 (LFD).

### 2.2. Follow-up protocol

To evaluate long-term outcomes, all participants completing the 6-week intervention and post-intervention assessments were invited to participate in a prospective follow-up. Additional assessments were scheduled at 6 months and 12 months post-intervention to capture clinical and microbiome trajectories. The follow-up protocol was harmonized with the original design to enable consistent within-subject comparisons.

#### 2.2.1. Eligibility for follow-up

Inclusion criteria for entry into the follow-up cohort were successful completion of the initial intervention phase, availability of baseline and immediate post-intervention data, willingness to provide follow-up stool samples and questionnaires, and absence at follow-up entry of major new dietary regimens, major gastrointestinal or systemic surgery, cancer therapy, immunosuppressive treatment, chronic infection management, recent gastrointestinal infection requiring antimicrobials (within 3 months), hospitalization (within 6 months), regular microbiome-modifying medication use, prebiotic/probiotic/synbiotic supplementation or excessive fermented-food intake (within 1–3 months), pregnancy or lactation, or inability to complete follow-up procedures.

These eligibility criteria were applied at entry to the follow-up cohort. Antibiotic, prebiotic, probiotic, and other microbiome-modifying exposures occurring during the subsequent non-interventional follow-up were recorded prospectively (Supplementary Tables S4 and S5) and treated as observed exposures rather than grounds for withdrawal or sample exclusion, so as not to bias this naturalistic long-term follow-up; their potential influence is considered in the Discussion.

#### 2.2.2. Clinical assessments

At each follow-up visit (6 and 12 months post-intervention), participants completed the same validated questionnaires used in the original RCT:

• **Irritable Bowel Syndrome Severity Scoring System (IBS-SSS):** Evaluates abdominal pain, bloating, bowel habits, and symptom interference (range 0–500; higher scores indicate greater severity).
• **Irritable Bowel Syndrome Quality of Life (IBS-QOL) Scale:** Assesses physical, emotional, and social well-being.
• **Hospital Anxiety and Depression Scale (HADS):** Screens for psychological distress via 14 items across two subscales (anxiety, depression). Higher scores indicate greater severity of symptoms.

### 2.3. Microbiome data collection and analysis

#### 2.3.1. Sample collection

At 6 and 12 months post-intervention, fecal samples were collected from all participants to assess long-term microbiome dynamics. Participants were provided with sterile collection kits and instructed to obtain a fresh stool sample at home. Samples were stored immediately at −20 °C in domestic freezers, transported to study centers within 24 hours on cold packs, and stored at −80 °C until processing, following the same standardized protocol as in the original RCT.

#### 2.3.2. DNA extraction and sequencing

Microbial DNA was extracted using the Qiagen PowerSoil DNA Isolation Kit (Qiagen, Hilden, Germany) according to manufacturer’s instructions. DNA concentration and purity were determined using the Qubit dsDNA HS Assay Kit and Qubit 2.0 fluorometer (Thermo Fisher Scientific, USA). The V4 hypervariable region of the 16S rRNA gene was amplified with primers 515F/806R, and sequencing libraries were prepared following the Illumina 16S Metagenomic Sequencing protocol. Libraries were pooled with a 15% PhiX spike-in and sequenced on an Illumina MiSeq platform (2 × 250 bp paired-end reads), targeting a minimum depth of 50,000 reads per sample.

#### 2.3.3. Bioinformatic processing

Raw sequencing reads were processed using the QIIME2 pipeline. Reads were quality-filtered (PHRED ≥ 30) and denoised using DADA2 to generate amplicon sequence variants (ASVs). Taxonomic classification was performed using a Naive Bayes classifier trained on the SILVA 132 reference database.

#### 2.3.4. Diversity analyzes

• Alpha diversity was assessed using Shannon index to evaluate within-sample richness and evenness.
• Beta diversity was calculated using Bray–Curtis dissimilarities, with group comparisons assessed by PERMANOVA (999 permutations). Principal coordinates analysis (PCoA) was used for visualization.

### 2.4. Patient and public involvement

Patients actively participated in the design and implementation of this research. During the feasibility stage, participants from the pilot clinical trial contributed feedback on recruitment strategies, study logistics, and participant experience with the personalized dietary approach, which informed refinements to the protocol and data collection tools. Their perspectives ensured that the study design addressed patient-relevant outcomes such as symptom burden, quality of life, and long-term feasibility of dietary interventions. Following publication, participants will receive a lay summary of the results through a study newsletter prepared for a nonspecialist audience, ensuring transparent communication and knowledge sharing with the IBS patient community.

### 2.5. Statistical analysis

Longitudinal changes were assessed using:

• **Linear mixed-effects models (LMM):** Diet group (PD vs. LFD), time (baseline, 6 weeks, 6 months, 12 months), and their interaction (group × time) were modeled as fixed effects, with subject ID as a random effect. This was the primary method to analyze continuous outcomes (IBS-SSS, IBS-QOL, HADS scores).
• **Repeated-measures ANOVA:** Conducted as a complementary method to assess within- and between-group changes over time. Greenhouse–Geisser correction was applied when sphericity assumptions were violated.
• Responder status was defined a priori as a reduction of at least 50 points in IBS-SSS from baseline. Responder proportions were compared between groups using Fisher exact tests, with Newcombe hybrid-score 95% confidence intervals for the between-group risk difference.
• Clinical outcomes and microbiome profiles were analyzed using endpoint-specific availability. Cross-sectional analyzes used all available observations for the relevant data domain, whereas the repeated-measures alpha-diversity ANOVA was restricted to participants with analyzable stool profiles at all four time points. Because questionnaire completion and stool-specimen provision were not identical, clinical, microbiome, and linked clinical-microbiome denominators are reported separately (Supplementary Table S6). Beta diversity was assessed using Bray-Curtis dissimilarities, with PERMANOVA performed separately at each time point using 999 permutations (pseudo-F, R^2^, and permutation *p* reported). PERMDISP was based on distances to group spatial medians with the small-sample bias adjustment and 9,999 residual permutations. At 6 and 12 months, PERMANOVA and PERMDISP used the same participants and the same Bray-Curtis matrices. The only recoverable 6-week PERMDISP output used the four-time-point complete-case subset (PD *n* = 39; LFD *n* = 31), rather than the full 6-week PERMANOVA set, and is reported only as a sensitivity analysis. The archived material did not contain the exact 6-week Bray-Curtis matrix or underlying abundance table needed to reconstruct a same-sample PERMDISP; accordingly, the full-sample 6-week PERMANOVA result is treated as exploratory and assumption-limited, and no centroid-separation inference is made.
• **Post-hoc analyzes:** Pairwise comparisons between groups and time points were performed, and *p*-values were adjusted for multiple testing using Holm–Bonferroni corrections.
• **Responder analyzes by baseline severity (mild, moderate, severe):** Proportions were summarized descriptively using the same ≥ 50-point IBS-SSS reduction threshold. Formal within-stratum tests were not performed because several cells were too small for reliable inference (Supplementary Table S8).
• **Attrition bias analysis:** To evaluate potential bias due to loss to follow-up, baseline and 6-week outcomes were compared between participants who completed vs. those who dropped out of the 12-month follow-up. Independent-sample t tests were used.
• **Effect sizes and significance:** Generalized eta-squared was reported for repeated-measures ANOVA models, and fixed-effect estimates were reported for LMMs. Statistical significance was defined as two-sided *α* = 0.05.
• The original randomized trial was prospectively powered for the 6-week primary endpoint as described in the parent publication. The present 12-month follow-up analyzes were not prospectively powered for between-group contrasts after accounting for observed attrition and should therefore be interpreted as long-term follow-up analyzes. Where appropriate, complementary analyzes specified for this follow-up (responder analyzes for IBS-SSS and longitudinal mixed-effects interaction tests for all clinical outcomes) were used to triangulate findings less sensitive to pairwise time-point sample size.
• Differential abundance analysis: To identify candidate taxonomic drivers underlying the observed microbiome changes, exploratory differential abundance analyzes were performed at the genus and family levels using the 12-month microbiome profiles. Relative abundances were log-transformed after addition of a small pseudocount to stabilize variance. Differences between PD and LFD groups were tested using the Mann–Whitney U test. Effect sizes were expressed as log10 fold-change of group means. *P*-values were adjusted for multiple comparisons using the Benjamini–Hochberg false discovery rate (FDR).
• Functional metagenome inference: Exploratory taxonomy-based functional proxy analysis: Because this study generated 16S rRNA amplicon data rather than shotgun metagenomic sequencing, microbial functional activity was not directly measured. To provide an exploratory, hypothesis-generating view of potential functional patterns, taxonomy-based proxy scores were calculated from the 16S taxonomic profiles. For each predefined functional category, short-chain fatty acid-associated taxa (including butyrate and propionate potential), lactate production/utilization, bile salt hydrolase (BSH)-associated taxa, mucin-associated taxa, methane-associated taxa, and sulfate/H₂S-associated taxa, the proxy score was defined as the summed relative abundance of bacterial taxa curated a priori as commonly associated with that function. These scores should be interpreted as approximate ecological indicators of taxonomic composition, not as gene-family, pathway-abundance, metabolite, or enzyme-activity measurements. PD and LFD groups were compared at 12 months using Mann-Whitney U tests with Benjamini-Hochberg FDR correction. Longitudinal trajectories of selected proxy scores were assessed using mixed-effects models with fixed effects for group, time, and group × time interaction and participant as a random intercept.
• Antibiotic-exposure assessment. Self-reported antibiotic use during follow-up was captured at the 6-month and 12-month visits using the structured questionnaire shown in Supplementary Table S4. Between-group comparisons of binary exposure used Fisher's exact test, with effect size reported as absolute risk difference (95% CI) and Cramér's V. Between-group comparisons of total duration of use among exposed participants used Welch's t-test.

**Specific Taxa Analysis**

Relative abundance of Faecalibacterium prausnitzii was selected as a taxonomic marker of interest given its relevance to gut health and anti-inflammatory activity. Changes over time and between groups were evaluated using both LMM and repeated-measures ANOVA.

All statistical analyzes were performed in Python 3.11.8 (statsmodels, pingouin, and scikit-bio), with the exception of the multivariate homogeneity-of-dispersion test (PERMDISP; betadisper procedure), which compared distances to each group’s spatial median in principal-coordinate space using 9,999 residual permutations with a small-sample bias adjustment. Significance was set at a two-sided *α* = 0.05, and effect sizes (η^2^ for ANOVA, fixed-effect estimates for LMM) were reported where applicable.

## 3. Results

### 3.1. Follow-up participation

At the 6-month follow-up, 58 patients in the PD group and 41 patients in the LFD group completed clinical assessments (microbiome profiles were available for 56 and 40, respectively). Attrition in the PD group (*n* = 12) was primarily due to relocation (*n* = 2) or discontinuation of participation (*n* = 10). In the LFD group (*n* = 10), reasons for attrition included relocation (*n* = 1), withdrawal (*n* = 6), and dissatisfaction with the intervention (*n* = 3).

**Figure 1.**
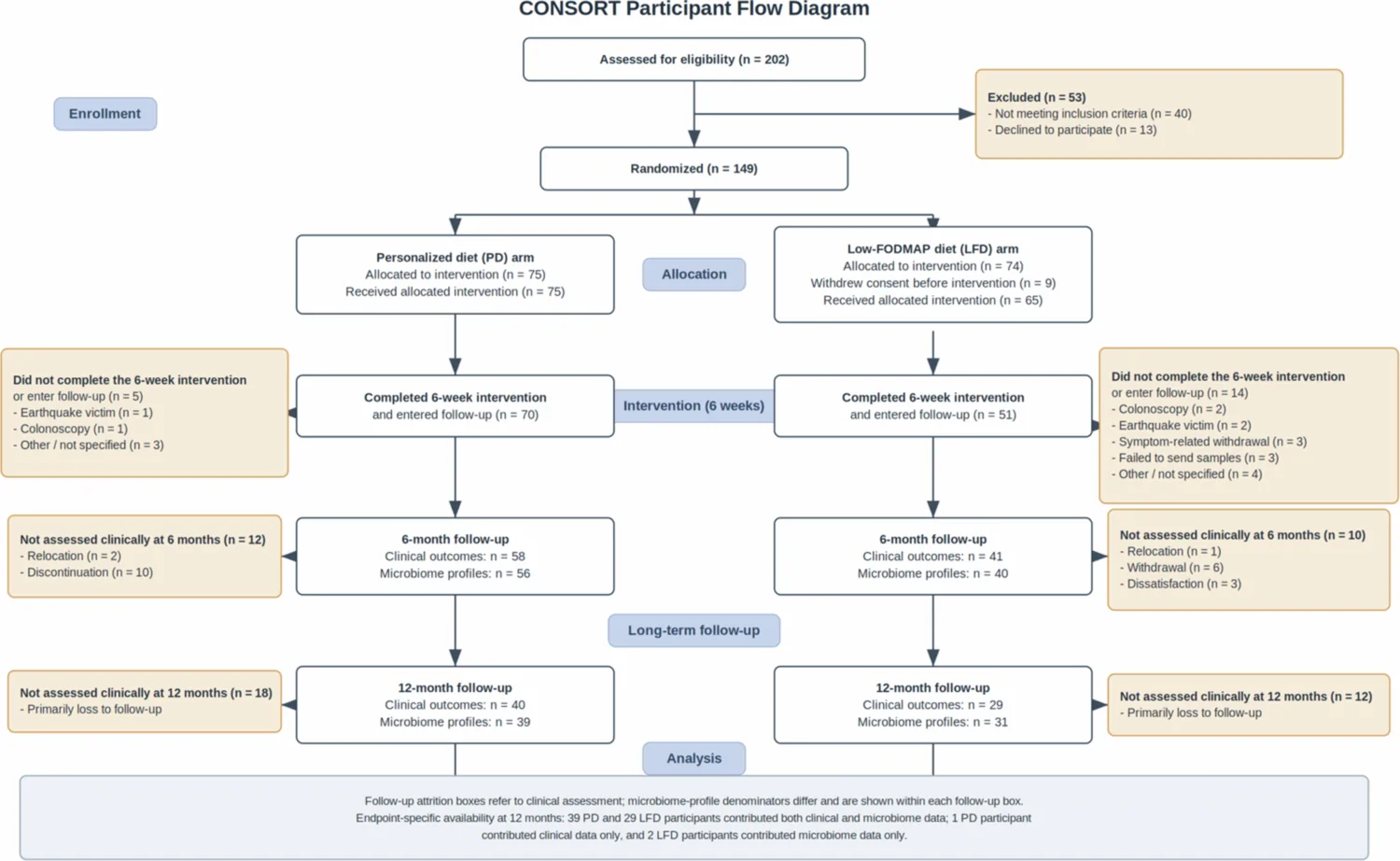
CONSORT diagram.

At the 12-month follow-up, 40 PD and 29 LFD participants completed the clinical assessment. Attrition between the 6- and 12-month assessments was higher, with 18 participants discontinuing in the PD group and 12 in the LFD group, mostly due to loss to follow-up. The overall participant flow, including enrollment, allocation, and endpoint-specific clinical and microbiome availability at each time point, is summarized in the CONSORT diagram (Figure 1).

Baseline demographic and clinical characteristics of participants who completed the initial 6-week intervention are presented in Table 1. No statistically significant differences were observed for age, sex, BMI, IBS subtype distribution, stool consistency, HADS-Anxiety, HADS-Depression, or IBS-QOL (all *p* > 0.05). IBS-C was the most prevalent subtype in both groups (PD 45.7%; LFD 47.1%), followed by IBS-M (31.4%; 33.3%) and IBS-D (22.9%; 19.6%). The IBS-SSS severity distribution was also comparable. Baseline IBS-SSS and stool frequency were higher in PD (IBS-SSS Welch *p* = 0.027; stool frequency Welch *p* = 0.002; individual-level Mann–Whitney sensitivity *p* = 0.058). These chance imbalances are reported transparently; the longitudinal results are expressed as changes from baseline and group × time contrasts, and possible regression to the mean remains a consideration.

**Table 1.** Baseline demographic and clinical characteristics of the analyzed cohort (participants who completed the 6-week intervention: PD, *n* = 70; LFD, *n* = 51). All values are measured at baseline.

Characteristic	PD group (*n* = 70)	LFD group (*n* = 51)	*P* value	Test
Age	35.94 ± 10.13	37.9 ± 9.87	0.288	Welch t
Sex (female), *n* (%)	42 (60)	31 (60.8)	0.931	χ^2^
BMI	25.20 ± 5.02	25.07 ± 4.59	0.883	Welch t
**IBS subtype, *n* (%)**			0.910	χ^2^
IBS-C	32 (45.7)	24 (47.1)		
IBS-M	22 (31.4)	17 (33.3)		
IBS-D	16 (22.9)	10 (19.6)		
IBS-SSS (total score)	314.41 ± 92.79	276.76 ± 90.15	**0.027**	Welch t
**IBS-SSS severity, *n* (%)^a^**			0.717	χ^2^
Mild	5 (7.1)	5 (9.8)		
Moderate	30 (42.9)	24 (47.1)		
Severe	35 (50.0)	22 (43.1)		
Stool frequency^b^	7.13 ± 4.81	5.09 ± 2.17	**0.002**	Welch t
Stool consistency^b^	3.43 ± 1.40	3.65 ± 1.45	0.405	Welch t
HADS (anxiety)	10.27 ± 4.22	10.74 ± 3.95	0.531	Welch t
HADS (depression)	7.57 ± 4.35	8.33 ± 4.36	0.345	Welch t
IBS-QOL score	45.55 ± 22.06	42.65 ± 19.82	0.450	Welch t

Data are mean ± SD or n (%). Continuous variables were compared using two-sample Welch t tests calculated from the displayed means, SDs, and group sizes; categorical variables used Pearson chi-square tests. IBS subtype and IBS-SSS severity each use one omnibus 2-df test; category rows show counts only.

^a^ For IBS-SSS severity, one expected cell is below 5; the non-significant Pearson chi-square result should therefore be interpreted cautiously.

^b^ Stool frequency is right-skewed. The table reports the reproducible Welch result from the displayed summary statistics (p = 0.002); the supplied individual-level Mann–Whitney sensitivity result was p = 0.058. Stool consistency is ordinal and its Welch comparison should be interpreted descriptively.

### 3.2. Long-term clinical outcomes

#### 3.2.1. IBS-SSS

**Figure 2.**
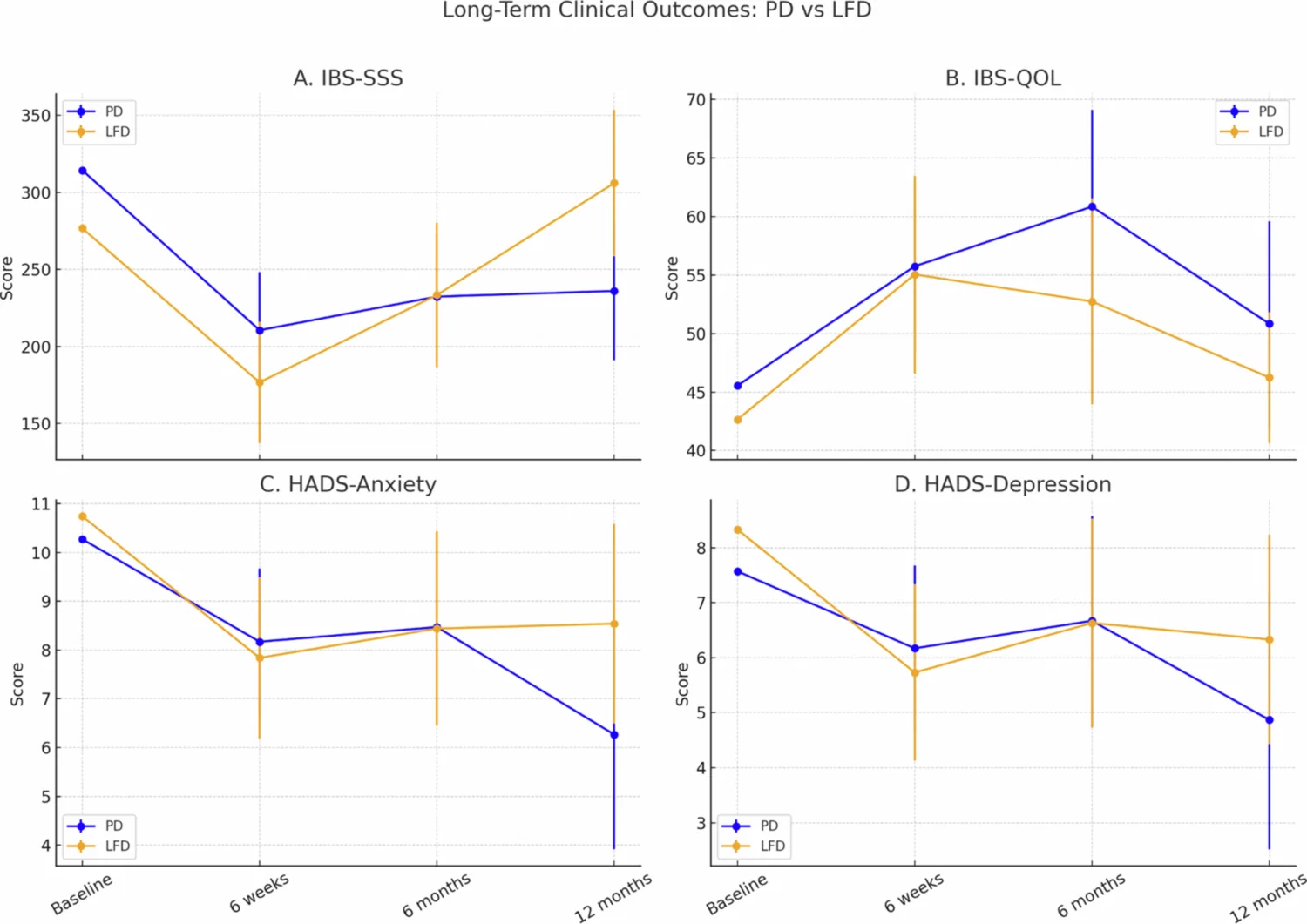
Group-specific clinical outcome trajectories for (A) IBS-SSS, (B) IBS-QOL, (C) HADS-Anxiety, and (D) HADS-Depression from baseline through 12 months. Points show group mean scores. At follow-up visits, vertical bars show the 95% confidence intervals for the reported changes from baseline translated to the score scale; baseline points are shown without uncertainty bars. PD, personalized diet; LFD, low-FODMAP diet; IBS-SSS, Irritable Bowel Syndrome Symptom Severity Score; IBS-QOL, Irritable Bowel Syndrome Quality of Life; HADS, Hospital Anxiety and Depression Scale.

Table 2 presents changes in symptom severity, quality of life, and psychological outcomes over 12 months. It reports group-specific mean changes with 95% confidence intervals, between-group *p* values for differences in change, and longitudinal group × time *p* values. Both PD and LFD reduced IBS-SSS at 6 weeks. Improvements persisted at 6 and 12 months in PD (−82.0 and −78.3 points), whereas LFD returned toward baseline at 12 months (+29.3 points; *p* = 0.001 for the between-group difference in change) (Figure 2). IBS-C and IBS-M showed the clearest long-term PD reductions; the IBS-D between-group difference was not statistically significant at 12 months. Responder rates were similar at 6 weeks (72.9% vs 72.5%; Fisher *p* = 1.000) and did not differ significantly at 6 months (56.9% vs 48.8%; *p* = 0.540). At 12 months, responder rates were 62.5% in PD and 34.5% in LFD, an absolute risk difference of +28.0 percentage points (95% CI 4.2 to 47.7; Fisher *p* = 0.029) (Table 3). Severity-stratified proportions are reported descriptively in Supplementary Table S8.

**Table 2.** Group-specific changes over time in IBS-SSS, IBS-QOL, and HADS outcomes, with between-group comparisons at 6 weeks, 6 months, and 12 months.

Variable	PD baseline mean ± SD	PD change 0–6 weeks (95% CI)	PD change 0–6 months (95% CI)	PD change 0–12 months (95% CI)	LFD baseline mean ± SD	LFD change 0–6 weeks (95% CI)	LFD change 0–6 months (95% CI)	LFD change 0–12 months (95% CI)	Between-group *p*, 6 weeks	Between-group *p*, 6 months	Between-group *p*, 12 months	Group × time p
IBS-SSS	314.41 ± 92.79	−103.8 (−141.3, −66.2)	−82.0 (−122.3, −41.6)	−78.3 (−123.1, −33.4)	276.76 ± 90.15	−100.0 (−139.2, −60.6)	−43.3 (−90.2, 3.5)	+29.3 (−18.1, 76.7)	0.891	0.220	0.001	<0.001
IBS-C	327.91 ± 97.74	−126.2 (−163.0, −89.5)	−75.6 (−113.4, −37.8)	−87.6 (−131.8, −43.4)	295.46 ± 85.94	−102.7 (−143.1, −62.3)	−40.5 (−79.9, −1.0)	+16.4 (−32.0, 64.8)	0.399	0.208	0.002	<0.001
IBS-D	306.06 ± 100.88	−84.9 (−122.8, −47.1)	−78.8 (−118.0, −39.5)	−15.2 (−63.1, 32.7)	223.08 ± 97.44	−52.3 (−92.6, −12.0)	+35.6 (−22.2, 93.4)	+37.1 (−25.0, 99.3)	0.248	0.001	0.191	0.141
IBS-M	300.86 ± 80.01	−113.5 (−152.9, −74.0)	−99.3 (−143.5, −55.0)	−100.6 (−144.3, −56.8)	290.71 ± 74.47	−132.9 (−167.5, −98.2)	−106.6 (−150.8, −62.4)	+30.3 (−6.2, 66.8)	0.469	0.819	< 0.001	<0.001
IBS-QOL	45.55 ± 22.06	+10.2 (3.0, 17.5)	+15.3 (7.0, 23.5)	+5.3 (−3.4, 14.1)	42.65 ± 19.82	+12.4 (4.0, 20.9)	+10.1 (1.3, 18.9)	+3.6 (−2.0, 9.2)	0.699	0.398	0.748	0.018
IBS-C	45.86 ± 22.25	+9.6 (2.0, 16.9)	+1.8 (−7.3, 10.4)	+3.0 (−7.8, 13.8)	41.36 ± 17.51	+17.0 (8.9, 25.1)	+12.6 (4.7, 20.6)	+13.2 (3.6, 22.9)	0.188	0.075	0.167	0.087
IBS-D	45.82 ± 25.21	+12.6 (4.8, 20.4)	+8.7 (0.3, 17.1)	+7.6 (−1.2, 16.4)	41.67 ± 20.90	+18.6 (10.0, 27.2)	+11.5 (2.9, 20.1)	+6.1 (−3.9, 16.0)	0.311	0.648	0.825	0.649
IBS-M	44.89 ± 20.35	+9.7 (2.0, 17.4)	+9.8 (1.6, 18.0)	+7.7 (−1.0, 16.3)	45.93 ± 21.96	+2.3	+11.7 (2.6, 20.8)	+1.1 (−8.3, 10.4)	—	0.761	0.310	0.352
HADS-Anxiety	10.27 ± 4.22	−2.1 (−3.6, −0.6)	−1.8 (−3.7,+0.2)	−4.0 (−6.3, −1.6)	10.74 ± 3.95	−2.9 (−4.5, −1.2)	−2.3 (−4.3, −0.3)	−2.2 (−4.3, −0.2)	0.482	0.726	0.258	<0.001
HADS-Depression	7.57 ± 4.35	−1.4 (−2.9,+0.1)	−0.9 (−2.8,+1.0)	−2.7 (−5.0, −0.3)	8.33 ± 4.36	−2.6 (−4.2, −1.0)	−1.7 (−3.6,+0.2)	−2.0 (−3.9, −0.1)	0.284	0.560	0.650	0.003

Values are baseline mean ± SD or mean change from baseline with 95% CI, except where marked unavailable. Change is follow-up minus baseline. Between-group p values are two-sided inverse-variance normal contrasts of the group-specific change estimates (ΔPD − ΔLFD); standard errors were derived from the corresponding 95% CIs and combined across the independent groups. These CI-derived p values are post hoc contrasts and are not reproductions of the parent trial’s separately reported between-group tests. Group × time p values test the longitudinal interaction. Negative changes indicate improvement for IBS-SSS and HADS, whereas positive changes indicate improvement for IBS-QOL. Standardized effects are not reported because endpoint-specific change-score SDs were unavailable for all subgroup estimates; unstandardized changes and 95% CIs are retained. Abbreviations: PD, personalized diet; LFD, low-FODMAP diet; CI, confidence interval; IBS-SSS, Irritable Bowel Syndrome Symptom Severity Score; IBS-QOL, Irritable Bowel Syndrome Quality of Life; HADS, Hospital Anxiety and Depression Scale.

**Table 3.** Responder analysis (≥50-point reduction in IBS-SSS from baseline).

Timepoint	PD responders, n/N (%)	LFD responders, n/N (%)	Risk difference, percentage points (95% CI, Newcombe)	Fisher exact *p*
6 weeks	51/70 (72.9)	37/51 (72.5)	+0.3 (−15.1, 16.5)	1.000
6 months	33/58 (56.9)	20/41 (48.8)	+8.1 (−11.4, 26.9)	0.540
12 months	25/40 (62.5)	10/29 (34.5)	+28.0 (4.2, 47.7)	0.029

Responder = reduction of at least 50 points in IBS-SSS from baseline, among participants with a valid score at that visit. Difference = PD% minus LFD%. CI by the Newcombe hybrid-score method. p by Fisher exact test (two-sided).

#### 3.2.2. IBS-QOL

Both interventions improved IBS-QOL scores at 6 weeks. The PD group demonstrated sustained, albeit modest, improvements through 12 months (+5.3 points), whereas the LFD group showed smaller long-term gains (+3.6 points). The longitudinal analysis specified for this follow-up showed a significant group × time interaction (*p* = 0.018), indicating divergent trajectories between the two diets. Pairwise between-group contrasts at individual follow-up time points did not reach statistical significance, which—given the reduced sample size at 12 months—should not be interpreted as evidence of equivalence between interventions.

#### 3.2.3. Psychological outcomes

HADS-Anxiety scores improved significantly in both groups at 6 weeks, with reductions persisting through 12 months. The PD group showed a greater decline at 12 months (−4.0 points) compared with the LFD group (−2.2 points), although between-group differences were not statistically significant. HADS-Depression scores also improved in both groups, with sustained decreases at 12 months (PD: −2.7; LFD: −2.0). A significant group × time interaction was observed for both anxiety (*p* < 0.001) and depression (*p* = 0.003), reflecting a stronger trajectory of improvement in the PD group.

### 3.3. Microbiome diversity analysis

At 6 months, clinical outcomes were available for 58 PD and 41 LFD participants and microbiome profiles for 56 PD and 40 LFD participants. At 12 months, clinical outcomes were available for 40 PD and 29 LFD participants and microbiome profiles for 39 PD and 31 LFD participants. At 12 months, 39 PD and 29 LFD participants had both data types; one PD participant contributed clinical data only and two LFD participants contributed microbiome data only. The repeated-measures alpha-diversity analysis included 39 PD and 31 LFD four-timepoint microbiome complete cases, accounting for the reported denominator degrees of freedom.

#### 3.3.1. Alpha diversity

Alpha diversity, estimated by the Shannon index, was analyzed using a linear mixed-effects model (LMM) to assess within-subject changes over time and between-group differences (PD vs. LFD). Baseline values for the PD group were coded as the reference, with additional fixed terms for Time (6 weeks, 6 months, 12 months), Group (LFD), and the Group × Time interaction. Random intercepts were included to account for repeated measures within individuals.

At baseline, no significant differences were observed between groups (estimate = 0.056, *p* = 0.501). Both interventions were associated with increases in alpha diversity over time; however, improvements were consistently greater in the PD group. In PD, alpha diversity rose significantly at 6 weeks (+0.488, *p* < 0.001), remained elevated at 6 months (+0.300, *p* < 0.001), and persisted above baseline at 12 months (+0.205, *p* = 0.008). By contrast, the LFD group exhibited smaller gains, as reflected in negative interaction terms (6 weeks: −0.296, *p* = 0.011; 6 months: −0.387, *p* = 0.001; 12 months: −0.233, *p* = 0.046) (Table 4).

**Table 4.** Linear mixed-effects model estimates for alpha diversity (Shannon index).

Coefficient	Estimate	Std. Error	z-value	*p*-value	95% CI
Intercept (PD at baseline)	3.610	0.063	56.874	<0.001	3.486, 3.734
LFD group at baseline	0.056	0.083	0.672	0.501	−0.107, 0.219
6 weeks (PD change from baseline)	0.488	0.078	6.274	<0.001	0.335, 0.640
6 months (PD change from baseline)	0.300	0.078	3.854	<0.001	0.147, 0.452
12 months (PD change from baseline)	0.205	0.078	2.637	0.008	0.053, 0.357
LFD × 6 weeks	−0.296	0.117	−2.534	0.011	−0.525, −0.067
LFD × 6 months	−0.387	0.117	−3.316	0.001	−0.616, −0.158
LFD × 12 months	−0.233	0.117	−1.995	0.046	−0.462, −0.004
Participant random-intercept variance	0.039	0.041	—	—	—

The model used the four-time-point microbiome complete-case set (PD n = 39; LFD n = 31) with a participant-level random intercept. PD at baseline is the reference. The LFD baseline coefficient is the between-group baseline difference; PD time coefficients are changes from baseline in PD; LFD × time coefficients are the additional changes in LFD relative to PD. The final row is the estimated participant random-intercept variance. CI, confidence interval.

Post hoc pairwise comparisons further confirmed no baseline differences (*t* = −0.44, df = 61.30, *p* = 0.664, *d* = -0.11). From 6 weeks onward, the PD group consistently showed significantly higher alpha diversity than LFD: at 6 weeks (*t* = 2.04, df = 52.22, *p* = 0.046, *d* = 0.51), at 6 months (*t* = 3.05, df = 61.93, *p* = 0.003, *d* = 0.74), and at 12 months (*t* = 2.12, df = 52.33, *p* = 0.039, *d* = 0.53). These effect sizes indicate a moderate separation between the dietary interventions.

**Figure 3.**
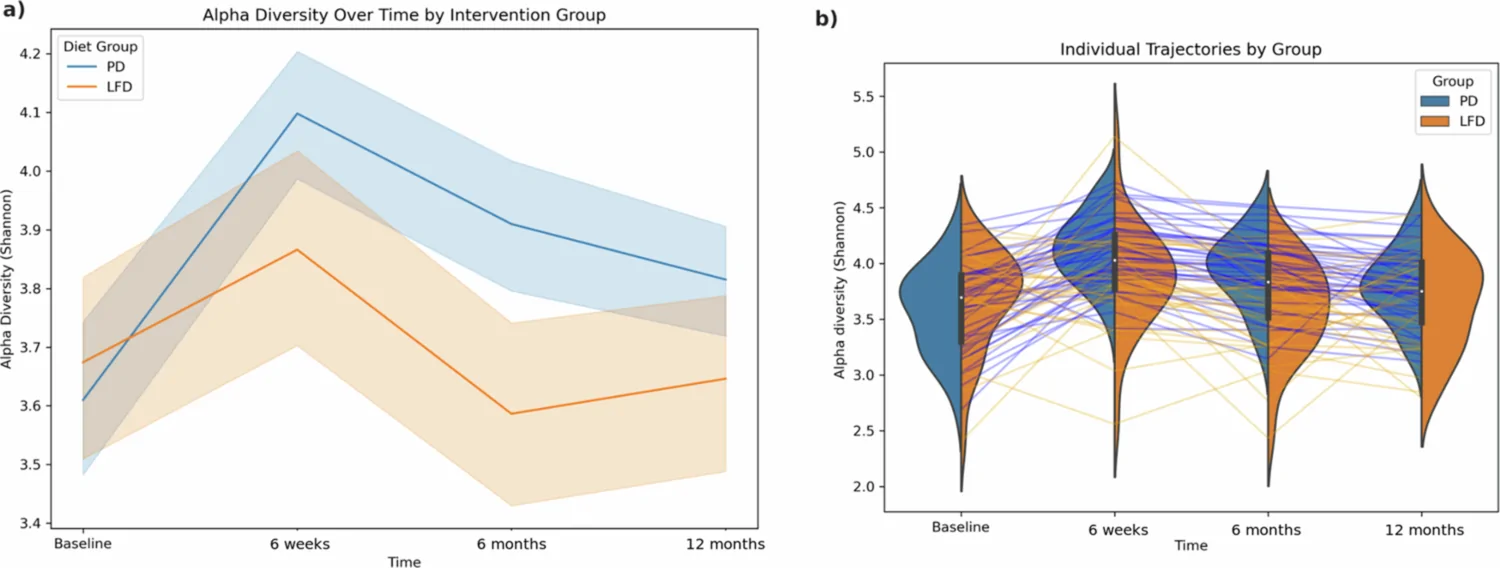
*(a)* Mean alpha diversity (Shannon index) with 95% confidence intervals for the PD and LFD groups at baseline, 6 weeks, 6 months, and 12 months. *(b)* Violin plots showing the full distribution at each time point, with thin lines connecting repeated observations from the same participant. Both panels use the four-time-point complete-case microbiome subset (PD *n* = 39; LFD *n* = 31). Note: the y-axis is truncated to display small differences in Shannon diversity; statistical comparisons are reported in the text. PD, personalized diet; LFD, low-FODMAP diet.

To illustrate temporal changes in alpha diversity, mean Shannon index values with 95% confidence intervals are shown in Figure 3a, while the full distribution of values with individual trajectories is presented in Figure 3b. Both groups demonstrated increases relative to baseline by 6 weeks, but the PD group exhibited a more pronounced and sustained elevation across the follow-up period.

Repeated-measures ANOVA showed significant within-group time effects in PD (F(3,114) = 20.16, *p* < 0.001, generalized η^2^ = 0.16) and LFD (F(3,90) = 3.39, *p* = 0.021, generalized η^2^ = 0.054). Together with the LMM interaction terms and time-specific pairwise comparisons, these findings are consistent with different Shannon-diversity trajectories between groups.

#### 3.3.2. Beta diversity

**Figure 4.**
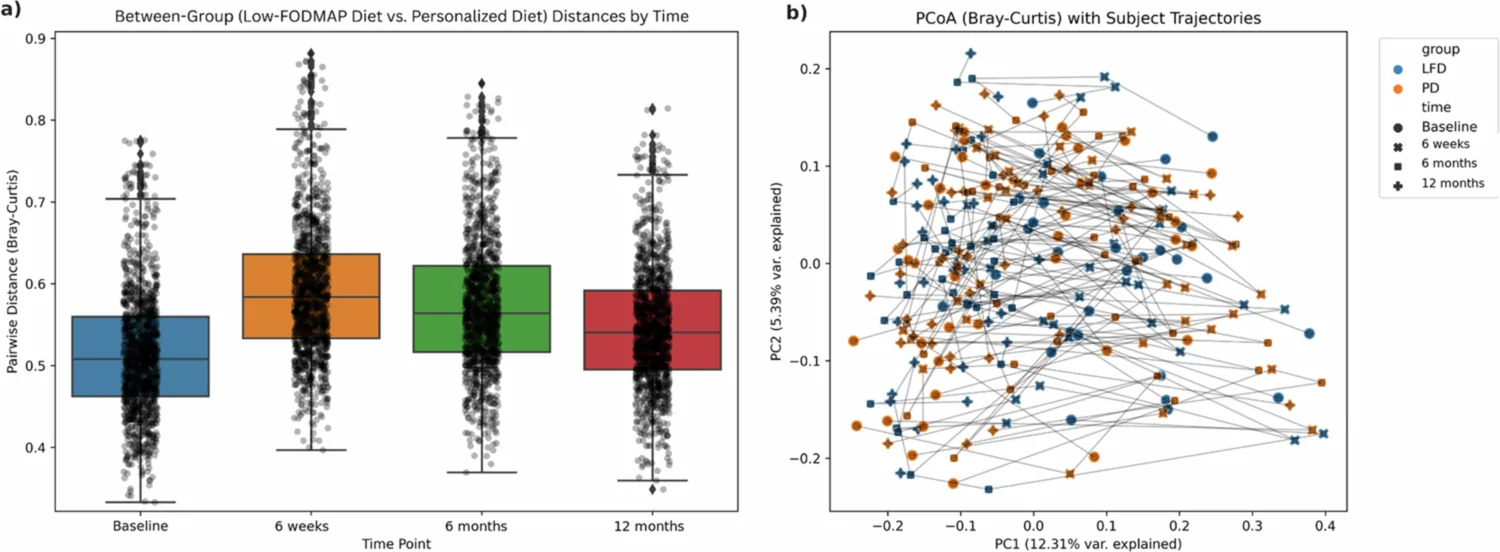
(a) Descriptive boxplots of pairwise between-group Bray-Curtis distances at baseline, 6 weeks, 6 months, and 12 months. Because pairwise distances are non-independent, these boxplots are descriptive and are not the basis of the PERMANOVA or PERMDISP *p* values. (b) Principal coordinates analysis (PCoA) of Bray-Curtis dissimilarities. Points are colored by diet group, symbols identify time points, and thin lines connect repeated samples from the same participant. Inferential PERMANOVA and PERMDISP results, including the assumption limitation at 6 weeks, are reported in the text and Supplementary Table S7. PD, personalized diet; LFD, low-FODMAP diet.

Beta diversity was assessed using PERMANOVA on Bray-Curtis dissimilarities to compare overall microbial community composition between PD and LFD at each time point. At baseline, no statistically significant between-group difference was detected (pseudo-F = 0.956, *p* = 0.414, R^2^ = 0.008). A nominal result was observed at 6 weeks (pseudo-F = 1.434, *p* = 0.042, R^2^ = 0.012). The difference was more pronounced at 6 months (pseudo-F = 3.411, *p* = 0.011, R^2^ = 0.035) and was not statistically significant at 12 months (pseudo-F = 1.494, *p* = 0.160, R^2^ = 0.0215) (Figure 4a). Same-sample PERMDISP with 9,999 residual permutations found no statistically significant dispersion difference at 6 months (PD *n* = 56; LFD *n* = 40; F(1,94) = 0.121, *p* = 0.733) or 12 months (PD *n* = 39; LFD *n* = 31; F(1,68) = 1.000, *p* = 0.319).

PCoA showed substantial overlap between groups. At 6 months, the modest PERMANOVA difference explained approximately 3.5% of Bray–Curtis variance and was not accompanied by a detectable same-sample dispersion difference. At 12 months, neither PERMANOVA nor PERMDISP was statistically significant.

#### 3.3.3. Candidate microbiome targets

An analysis of Faecalibacterium prausnitzii relative abundance showed no robust between-group separation over follow-up. In the longitudinal mixed-effects model, there was no strong baseline difference between groups (*β* = −0.021, *p* = 0.094), no clear PD time effects vs baseline (all *p* ≥ 0.454), and no statistically significant group × time interactions (largest at 12 months: *p* = 0.095). Between-group comparisons at each time point were also not significant (all *p* ≥ 0.21), indicating small effect sizes. Exploratory family-level differential abundance analysis at 12 months identified candidate family-level differences between PD and LFD; ranked family-level results, the volcano plot, and boxplots of selected families are provided in Supplementary Table S1 and Supplementary Figures S1 and S2. Corresponding genus-level results and longitudinal trajectories of selected genera are shown in Supplementary Table S2 and Supplementary Figure S3. Because shotgun metagenomic sequencing was not performed, microbial gene families, pathways, enzyme activities, and fecal metabolites were not measured directly. Exploratory taxonomy-based functional proxy scores were therefore treated only as hypothesis-generating ecological indicators. No PD–LFD contrast at 12 months reached FDR significance (Supplementary Table S3). Longitudinal plots of butyrate-, BSH-, sulfate/H₂S-, and lactate-associated proxy scores are provided in Supplementary Figures S4–S7.

### 3.4. Attrition bias analysis

Table 5 presents the comparison of baseline and 6-week outcomes between patients who completed the 12-month follow-up and those who dropped out. Analyzes included mean scores (±SD) for IBS-SSS, IBS-QOL, and Hospital Anxiety and Depression Scale (HADS) subscales, tested separately in the PD and LFD groups. Independent sample comparisons were performed at both baseline and 6 weeks.

**Table 5.** Baseline and 6-week outcomes in 12-month non-completers and completers (attrition analysis).

Group	Baseline non-completers, mean ± SD	Baseline completers, mean ± SD	*p*, baseline	6-week non-completers, mean ± SD	6-week completers, mean ± SD	*p*, 6 weeks
PD—IBS-SSS	310.6 ± 99.1	314.1 ± 86.1	0.875	227.7 ± 135.3	182.1 ± 125.1	0.154
LFD—IBS-SSS	274.7 ± 76.5	289.1 ± 94.8	0.534	186.6 ± 91.5	169.4 ± 125.0	0.573
PD—QOL	49.7 ± 22.8	44.8 ± 21.7	0.342	55.9 ± 18.2	56.1 ± 24.1	0.976
LFD—QOL	42.7 ± 22.8	41.9 ± 16.4	0.880	59.9 ± 22.6	51.4 ± 23.7	0.192
PD—Anxiety	9.8 ± 4.4	10.5 ± 4.2	0.510	9.0 ± 3.3	7.6 ± 3.8	0.113
LFD—Anxiety	10.3 ± 3.5	11.2 ± 4.3	0.403	7.9 ± 4.1	7.9 ± 4.2	0.999
PD—Depression	7.3 ± 4.6	7.8 ± 3.9	0.590	6.7 ± 4.6	5.9 ± 3.7	0.402
LFD—Depression	7.9 ± 5.1	8.7 ± 3.7	0.545	5.4 ± 4.2	6.1 ± 4.5	0.558

Note. Twelve-month non-completer/completer counts were PD 30/40 and LFD 22/29. Comparisons used independent-sample t tests within each intervention group. No comparison reached p < 0.05; these analyzes did not detect differences in the measured baseline or early outcomes, but limited power and unmeasured determinants of attrition mean that attrition bias cannot be excluded.

At baseline, IBS-SSS scores were similar between completers and drop-outs in both the PD group (314.1 ± 86.1 vs. 310.6 ± 99.1, *p* = 0.875) and the LFD group (289.1 ± 94.8 vs. 274.7 ± 76.5, *p* = 0.534). Likewise, QOL, anxiety, and depression scores did not differ significantly between completers and drop-outs (all *p* > 0.05).

At 6 weeks, no statistically significant differences were observed in symptom severity or anxiety and depression outcomes. In PD, mean IBS-SSS was 182.1 ± 125.1 in completers versus 227.7 ± 135.3 in non-completers (*p* = 0.154), and IBS-QOL was 56.1 ± 24.1 versus 55.9 ± 18.2 (*p* = 0.976). Anxiety and depression comparisons were also non-significant (*p* = 0.113 and *p* = 0.402). In LFD, completers and non-completers did not differ significantly in IBS-SSS (169.4 ± 125.0 vs 186.6 ± 91.5, *p* = 0.573), IBS-QOL (51.4 ± 23.7 vs 59.9 ± 22.6, *p* = 0.192), anxiety (7.9 ± 4.2 vs 7.9 ± 4.1, *p* = 0.999), or depression (6.1 ± 4.5 vs 5.4 ± 4.2, *p* = 0.558).

Taken together, these comparisons found no clear evidence that attrition was associated with the measured baseline characteristics or early treatment response. Residual attrition bias related to unmeasured factors cannot be excluded.

### 3.5. Follow-up dietary adherence and microbiome-modifying exposures

Self-reported dietary adherence and potential microbiome-modifying exposures during follow-up were captured at the 6-month and 12-month visits using a structured questionnaire (Supplementary Table S4), with antibiotic exposure summarized separately in Supplementary Table S5 given its recognized relevance to gut microbiome composition.

Dietary adherence was broadly comparable at 6 months (exact conditional *p* = 0.118); at 12 months the adherence distribution differed between groups, with better-preserved adherence in the PD group (exact conditional *p* = 0.010; small cell counts, interpret cautiously). Self-reported antibiotic use during the preceding 6 months was 43.1% (25/58) in the PD group versus 31.7% (13/41) in the LFD group at the 6-month visit (Fisher's exact *p* = 0.297; Cramér's V = 0.115), and 35.0% (14/40) versus 31.0% (9/29) at 12 months (*p* = 0.800; Cramér's V = 0.042); duration of use among exposed participants was comparable (6 months: 13.0 ± 13.2 vs 11.0 ± 8.5 days, Welch's t-test *p* = 0.576; 12 months: 9.5 ± 3.9 vs 13.0 ± 10.2 days, *p* = 0.349). Probiotic use was similar between arms (13.8–20.0% in PD, 17.1–17.2% in LFD; *p* ≥ 0.78), as was prebiotic use (3.4–19.5%; *p* ≥ 0.39). Travel, hospitalization, surgery, colonoscopy, and acute illness (including COVID-19) were similarly distributed between groups at both time points (all *p* ≥ 0.076); self-reported disease-status change at 12 months favored the PD group (exact conditional *p* = 0.020). Follow-up exposures are detailed in Supplementary Tables S4 and S5. These comparisons are exploratory and unadjusted for multiplicity.

## 4. Discussion

In this 12-month follow-up of an RCT restricted to participants who completed the initial 6-week intervention, the microbiome-guided personalized diet (PD) was associated with more durable IBS-SSS improvement than the low-FODMAP diet (LFD). Participants on PD maintained IBS-SSS improvements through one year and showed a more favorable IBS-QOL trajectory over time, supported by a statistically significant group × time interaction in the longitudinal mixed-effects analysis, although pairwise contrasts at individual time points did not reach significance given the reduced 12-month sample size. By contrast, LFD participants experienced regression of early symptom gains by 12 months. The PD arm also showed a sustained increase in alpha diversity from 6 weeks onward. In beta-diversity analyzes, a modest difference in Bray–Curtis composition was statistically detectable at 6 months but not at 12 months, with small PERMANOVA R^2^ values. These associations were observed across IBS subtypes (most consistently in IBS-C and IBS-M). Taken together, the findings are hypothesis-supporting: within the constraints of an open-label follow-up of 6-week completers, a microbiome-tailored nutritional approach was associated with more durable symptom control than LFD, a signal that requires confirmation in a prospectively powered long-term trial. Responder analyzes were consistent with this pattern, with a higher proportion of PD participants achieving a clinically meaningful IBS-SSS improvement at 12 months than LFD participants.

Dietary management is a cornerstone of IBS therapy, and contemporary guidelines endorse the LFD as an effective intervention, especially in the short term. The American College of Gastroenterology recommends a time-limited trial of a low-FODMAP diet to improve global IBS symptoms.^16^ Similarly, the British Society of Gastroenterology (BSG) includes the LFD as a second-line dietary therapy within a structured, dietitian-led care pathway.^17^ These recommendations reflect consistent evidence that LFD can alleviate overall IBS symptoms, abdominal pain, and bloating in the short run. A recent network meta-analysis of 13 RCTs (944 patients) confirmed that the LFD provides superior short-term relief: LFD ranked first among dietary interventions for improving global IBS symptoms, abdominal pain, and bloating, outperforming standard dietary advice.^18^ However, that meta-analysis also highlighted important gaps—most trials were in specialist centers with follow-up only to 4–12 weeks, and few assessed the effects of reintroducing FODMAPs or longer-term outcomes. In other words, while LFD’s short-term efficacy is well-established, its long-term durability and optimal personalization (after the elimination phase) have remained uncertain in the literature.^18^ Our 12-month findings extend this evidence base by directly addressing those gaps. We demonstrate that although LFD delivered initial symptom improvements (consistent with prior studies), these improvements were less consistently sustained at one year without ongoing personalization, whereas the microbiome-tailored PD both maintained clinical gains and was associated with sustained higher alpha diversity over the follow-up period, reported here as an ecological observation rather than as evidence of a “healthier” microbiome.

Emerging long-term data on the LFD have been mixed but instructive. For example, in one 12-month follow-up study of patients who underwent structured FODMAP reintroduction, about 67% reported adequate symptom relief at one year, and importantly, luminal *Bifidobacteria* abundances had returned to baseline levels after reintroducing higher-FODMAP foods.^19^ While personalization phases can partially restore beneficial taxa such as bifidobacteria after strict LFD, long-term follow-up data indicate that microbial metabolic shifts may persist, with fecal SCFA levels remaining below baseline despite symptomatic improvement.^19^ This underscores that simply liberalizing an LFD may not fully restore all facets of microbial function. Long-term adherence to LFD is variable, with reported rates ranging from ~50% to 82% and satisfaction at 70–89%. Barriers such as cost and social restrictions (e.g., eating out, travel) limit its practicality, underscoring why durable alternatives like PD may offer broader applicability.^20^ Against this backdrop, our study’s PD approach—which emphasizes inclusion of diverse, tolerated fibers rather than broad restriction—appears to offer a more sustainable path to symptom control, as reflected in the maintained improvements at 12 months.

From a pathophysiological standpoint, the results are compatible with models of IBS that include microbiome-related mechanisms,^21-24^ but they do not establish mediation. The LFD and PD represent different dietary strategies: LFD reduces fermentable substrates, whereas the personalized diet emphasizes diverse tolerated fibers.^25-30^ In this trial, the PD arm showed a persistent increase in Shannon alpha diversity. This is an ecological observation that is compatible with, but does not establish, a microbiome-mediated mechanism for durable clinical benefit.

This study provides uncommon 12-month clinical and microbiome follow-up after a randomized dietary intervention and includes several IBS subtypes. Validated clinical measures and longitudinal mixed-effects models were used to characterize trajectories. The coexistence of durable symptom improvement and persistent alpha-diversity differences provides preliminary, hypothesis-generating evidence for microbiome-informed dietary therapy, but does not establish a causal microbiome mechanism. A potential limitation relates to differences in perceived personalization between interventions. Participants receiving microbiome-guided recommendations may have experienced greater engagement or motivation than those following standardized dietary advice. Although counseling time and dietitian contact were standardized across groups, expectation or attention effects cannot be excluded.

Attrition over 12 months was substantial. The original randomized trial was prospectively powered for its 6-week primary endpoint and was not prospectively powered for 12-month between-group comparisons after observed attrition. Comparisons found no statistically significant baseline or 6-week differences between 12-month completers and non-completers in either group, but these analyzes have limited power and cannot exclude attrition related to unmeasured factors. For secondary outcomes, significant longitudinal interaction tests should be interpreted alongside non-significant pairwise contrasts and reduced long-term sample sizes.

A further limitation relates to antibiotic exposure during follow-up. Self-reported antibiotic use was common in both groups and may have influenced microbiome composition. Exposure was numerically higher in PD at 6 months but not statistically significantly different, and rates were similar at 12 months (Supplementary Table S5). Although residual confounding cannot be excluded, the small between-group differences in exposure—together with the consistency of the change-score and responder analyzes—make it unlikely that differential antibiotic exposure fully explains the principal clinical results. The microbiome interpretation is necessarily more cautious: exposure was captured by self-report without structured data on antibiotic class, indication, or timing relative to stool sampling. The follow-up assessment also lacked 3-day food diaries, food-frequency questionnaires, and prokinetic-medication capture. Residual confounding from unquantified habitual intake, adherence, and microbiome-modifying exposures cannot be excluded.

A further limitation is that PERMANOVA effect sizes were modest, with diet group explaining at most 3.5% of Bray-Curtis variance at 6 months and less at 6 weeks and 12 months. Same-sample PERMDISP using 9,999 residual permutations was non-significant at 6 months (F(1,94) = 0.121, *p* = 0.733) and 12 months (F(1,68) = 1.000, *p* = 0.319). The available 6-week PERMDISP result (F(1,68) = 1.83, *p* = 0.178) used only the four-time-point complete-case subset (PD *n* = 39; LFD *n* = 31), rather than the full 6-week PERMANOVA sample, and therefore cannot exclude a dispersion contribution to the full-sample result. The 6-month finding is consistent with a modest location difference without detectable unequal dispersion, although a dispersion contribution cannot be excluded definitively. Functional interpretation is also limited by 16S rRNA sequencing. Taxonomy-based functional proxies do not capture strain-level gene content, gene expression, enzyme activity, or metabolite concentrations and should not be interpreted as direct functional measurements.

For clinicians, the central implication of this follow-up is that personalization may matter for durability. A strict low-FODMAP diet without structured adaptation may show diminishing efficacy over time and could introduce nutritional imbalances, whereas a microbiome-guided personalized diet was associated with more sustained symptom relief alongside preserved microbial diversity in this cohort. These observations are consistent with a broader move in IBS dietary management away from uniform restriction toward biologically informed personalization, but they remain hypothesis-generating. Whether precision strategies grounded in diet–microbiome–host interactions can improve long-term IBS management will need to be established in dedicated, prospectively powered trials.

## 5. Conclusion

In summary, this 12-month follow-up of an RCT, restricted to participants who completed the 6-week intervention, found that a microbiome-guided personalized diet was associated with more durable improvements in IBS symptoms and with persistent alpha-diversity differences and modest 16S-based compositional differences, whereas the clinical benefits of a standard low-FODMAP diet diminished over time. Both dietary strategies were effective in the short term, consistent with guideline recommendations. The study also provides relatively uncommon long-term data on the low-FODMAP diet, suggesting that initial relief may regress in the absence of ongoing personalization and structured reintroduction; because the LFD comparator here did not include continued structured reintroduction, these results should not be generalized to the full contemporary low-FODMAP care pathway. Higher alpha diversity is reported as an ecological observation and is not equated with a “healthier” microbiome. Overall, these hypothesis-supporting findings warrant confirmation in larger trials prospectively powered for long-term endpoints before microbiome-informed nutrition is integrated into routine IBS management pathways.

## Supplementary Material

Supplementary Materials Revised.docx

## Data Availability

The 16S rRNA gene sequencing data generated and analyzed during the present study have been deposited in the NCBI BioProject database under accession number PRJNA1470994. The corresponding raw sequence reads have been deposited under this BioProject accession and will be released publicly through the NCBI Sequence Read Archive upon publication. De-identified aggregate results supporting the clinical and microbiome analyzes reported in this manuscript, including clinical outcome summaries, microbiome diversity metrics, taxonomic relative-abundance summaries, and follow-up exposure/adherence summaries, are available within the article and its supplementary materials. Individual-level raw clinical records and any data elements that could compromise participant privacy are not publicly available because participants did not provide consent for unrestricted sharing of identifiable or potentially re-identifiable health data. Requests for additional restricted de-identified data may be directed to the corresponding author and will be considered subject to ethics approval, institutional data-sharing agreements, and applicable data-protection regulations.

## Reporting guidelines

Reporting followed the CONSORT 2010 guidance for randomized trials; a completed CONSORT 2010 checklist is provided as a Supplementary file, and the participant-flow diagram is presented in Figure 1.
