# Supplementary material for: Long-term microbiome and clinical effects of a microbiome-guided personalized diet versus low-FODMAP diet in irritable bowel syndrome: A 12-month follow-up randomized controlled trial
Source: Gut Microbes. 2026 Aug 20;18(1):2719125. doi: 10.1080/19490976.2026.2719125 (PMC13502004; doi:10.1080/19490976.2026.2719125)
Supplement: Supplementary file 1 [file KGMI_A_2719125_SM6453.docx]

### Supplementary Materials

### Long-term Microbiome and Clinical Effects of a Microbiome-Guided Personalized Diet versus Low-FODMAP Diet in Irritable Bowel Syndrome: A 12-Month Follow-Up Randomized Controlled Trial

### Varol TUNALI¹^,^², Naciye Çiğdem ASLAN^3^, Gözde Derviş HAKİM^4^, Aycan GÜNDOĞDU^5^, Beyza Hilal ERMİŞ^6^, Mehmet HORA⁷, Özkan Ufuk NALBANTOĞLU⁸

¹ Department of Microbiology, Faculty of Medicine, Izmir University of Economics, Izmir, Turkey

² Department of Medical and Surgical Sciences, UOC Gastroenterologia, Fondazione Policlinico Universitario A. Gemelli IRCCS, Rome, Italy

^3^ Department of Gastrointestinal Surgery, Medipol Bahçelievler Hospital, Istanbul, Turkey

^4^ Department of Gastroenterology, Tepecik Education and Research Hospital, Izmir, Turkey

^5^ Department of Immunology, Faculty of Medicine, Medeniyet University, İstanbul, Turkey

^6^ Faculty of Nutrition and Dietetics, Adnan Menderes University, Aydın, Turkey

⁷ Bioinformatics Division, Genome and Stem Cell Center, Erciyes University, Kayseri, Turkey

⁸ Department of Computer Engineering, Faculty of Engineering, Dokuz Eylül University, Izmir, Turkey

Corresponding author
Varol TUNALI

Email: [varoltunali@gmail.com](mailto:varoltunali@gmail.com)

Address: Fevzi Çakmak, Sakarya Cd. No:156, 35330 Balçova, İzmir, Türkiye

**Supplementary File S1.** Example of a personalized microbiome-guided diet plan provided to participants in the PD group.

BREAKFAST (09:00–10:00)

3 DAYS PER WEEK

1 egg (boiled or omelet)

30 g full-fat white cheese / tofu cheese / Ezine cheese (about two finger-width in size) (sprinkle a pinch of black cumin seeds on top)

1 portion of fruit

1 cucumber

4–5 slices of peeled tomato

1 mild green pepper

1 mild red pepper (you may roast it in a toaster if desired)

1 carrot or grated carrot

7 raw almonds / 2 raw walnuts

2 thin slices of buckwheat bread

2 DAYS PER WEEK

2 thin slices of buckwheat bread

60 g aged kaşar cheese

Half a mild red pepper

1 teaspoon sesame seeds

You may consume this as a toast or cold sandwich. If the bread slices are small, you may consume 2 pieces. Additionally, consume 1 portion of fruit.

2 DAYS PER WEEK

1 glass of soy milk

4–5 tablespoons of buckwheat flakes

1 portion of fruit

7 raw almonds / 7 raw hazelnuts / 2 raw walnuts

1 teaspoon tahini

You may cook the milk and flakes on the stove before consuming.

SNACK (15:00)

1 cup mint tea

1 portion of fruit

7 raw almonds + 7 raw hazelnuts

DINNER (18:00–19:00)

3 DAYS PER WEEK

8 tablespoons olive-oil vegetable dish

4 tablespoons full-fat yogurt / homemade yogurt

2 thin slices of buckwheat bread

2 DAYS PER WEEK

150 g grilled / boiled / sautéed / baked red meat

6 tablespoons vegetable dish / sautéed vegetables / boiled vegetables / baked vegetables

4 tablespoons full-fat yogurt / homemade yogurt

2 thin slices of buckwheat bread

1 DAY PER WEEK

150 g grilled / boiled / sautéed / baked chicken

6 tablespoons vegetable dish / sautéed vegetables / boiled vegetables / baked vegetables

4 tablespoons full-fat yogurt / homemade yogurt

2 thin slices of buckwheat bread

1 DAY PER WEEK

200 g grilled / baked fish

6 tablespoons vegetable dish / sautéed vegetables / boiled vegetables / baked vegetables

1 thin slice of buckwheat bread

SNACK (21:00)

1 cup fennel tea
Note:

This document presents representative examples of dietary recommendations provided to participants during the intervention. Because all participants in this study were recruited in Türkiye, the original dietary recommendations were prepared and delivered in Turkish. The versions shown here represent English translations prepared for supplementary reporting purposes.

**Supplementary File S2.** Example of a low-FODMAP diet plan provided to participants in the LFD group.
FIRST MEAL

2 DAYS PER WEEK

1 egg (boiled or omelet)

30 g cheese (cheddar or cottage cheese)

1 portion fruit (grapes / kiwi / orange / pineapple / papaya / mandarin)

4–5 slices tomato

1 cucumber

1 carrot or grated carrot

5 black olives + 5 green olives

2 thin slices sourdough bread or gluten-free bread

1 DAY PER WEEK

2 thin slices sourdough bread or gluten-free bread

30 g cheese

1–2 slices tomato

1 cucumber or radish

1 teaspoon sesame seeds or olives

Consume as toast or sandwich. Additionally, eat 1 portion fruit (kiwi, grapes, orange, or pineapple).

1 DAY PER WEEK

1 cup almond milk / hemp milk

4–5 tablespoons oatmeal

1 portion fruit (banana – unripe / kiwi / grapes)

1 teaspoon flaxseed

1 DAY PER WEEK

12 tablespoons chicken vegetable dish

(vegetables may include carrot, spinach, tomato, eggplant, kale, bok choy)

4 tablespoons milk yogurt or coconut yogurt

2 thin slices sourdough bread

1 DAY PER WEEK

150 g grilled / boiled / sautéed beef

6 tablespoons vegetables (carrot, eggplant, spinach, kale, bok choy, tomato)

4 tablespoons milk yogurt or cottage cheese

2 thin slices sourdough bread

1 DAY PER WEEK

150 g grilled / boiled / sautéed chicken

6 tablespoons vegetables (spinach, carrot, tomato, eggplant)

4 tablespoons milk yogurt

2 thin slices sourdough bread

SNACK

1 cup tea or coffee

1 portion fruit (orange / kiwi / grapes / pineapple)

1 handful almonds

DINNER

3 DAYS PER WEEK

8 tablespoons olive oil vegetable dish

(eggplant, carrot, spinach, tomato, bok choy, kale)

4 tablespoons milk yogurt or coconut yogurt

1 thin slice sourdough bread

2 DAYS PER WEEK

150 g grilled beef

6 tablespoons vegetables (spinach, carrot, tomato, eggplant)

4 tablespoons milk yogurt

1 thin slice sourdough bread

1 DAY PER WEEK

120 g grilled chicken

6 tablespoons vegetables (bok choy, carrot, spinach)

4 tablespoons milk yogurt

1 thin slice sourdough bread

1 DAY PER WEEK

150 g grilled fish/seafood

6 tablespoons vegetables (spinach, kale, carrot, tomato)

1 thin slice sourdough bread

Note: This document presents representative examples of dietary recommendations provided to participants during the intervention. Because all participants in this study were recruited in Türkiye, the original dietary recommendations were prepared and delivered in Turkish. The versions shown here represent English translations prepared for supplementary reporting purposes.

### Supplementary Table S1. Top family-level differences at 12 months (ranked by p)

| **Family** | **Mean(PD)** | **Mean(LFD)** | **log10FC(PD/LFD)** | **p** | **q(FDR)** |
| --- | --- | --- | --- | --- | --- |
| Unclassified_Streptophyta | 3.09e-05 | 5.03e-05 | -0.207 | 0.0018 | 0.101 |
| Coriobacteriaceae | 0.00282 | 0.00214 | 0.12 | 0.0385 | 0.732 |
| Synergistaceae | 0.000694 | 2.26e-06 | 2.33 | 0.0515 | 0.732 |
| Bifidobacteriaceae | 0.00275 | 0.00159 | 0.238 | 0.0546 | 0.732 |
| [Odoribacteraceae] | 0.0135 | 0.00619 | 0.339 | 0.105 | 0.732 |
| Enterococcaceae | 0.000127 | 0.000116 | 0.0371 | 0.115 | 0.732 |
| unclassified_Synergistales | 0.000146 | 0.000102 | 0.154 | 0.124 | 0.732 |
| Enterobacteriaceae | 0.00304 | 0.0105 | -0.539 | 0.127 | 0.732 |
| Prevotellaceae | 0.167 | 0.216 | -0.111 | 0.159 | 0.732 |
| Dethiosulfovibrionaceae | 7.37e-06 | 1.49e-05 | -0.279 | 0.161 | 0.732 |
| Planococcaceae | 2.56e-05 | 1.5e-05 | 0.22 | 0.169 | 0.732 |
| [Tissierellaceae] | 0.00238 | 0.000457 | 0.717 | 0.187 | 0.732 |
| Anaeroplasmataceae | 0.00396 | 0.000113 | 1.54 | 0.191 | 0.732 |
| Peptococcaceae | 0.000197 | 0.000504 | -0.406 | 0.196 | 0.732 |
| unclassified_unknown | 0.000527 | 0.00107 | -0.307 | 0.231 | 0.732 |

**Figure S1.** Family-level volcano plot comparing PD and LFD groups at 12 months. Volcano plot illustrating differential abundance of bacterial families between the personalized diet (PD) and low-FODMAP diet (LFD) groups at the 12-month follow-up. Each point represents a bacterial family. The x-axis shows the log10 fold change in mean relative abundance between groups (log10[mean PD / mean LFD]), where positive values indicate higher abundance in the PD group and negative values indicate higher abundance in the LFD group. The y-axis represents the −log10(p value) from the Mann–Whitney U test. The dashed horizontal line indicates the nominal significance threshold (p = 0.05). Selected families with the largest differences are labeled for visualization.

### Supplementary Table S2. Top genus-level differences at 12 months (ranked by p)

| **Genus** | **Mean(PD)** | **Mean(LFD)** | **log10FC(PD/LFD)** | **p** | **q(FDR)** |
| --- | --- | --- | --- | --- | --- |
| Finegoldia | 2.77e-05 | 0.000109 | -0.585 | 0.0351 | 0.937 |
| unclassified_[Paraprevotellaceae] | 0.000139 | 0.000441 | -0.499 | 0.0458 | 0.937 |
| Bifidobacterium | 0.00275 | 0.00156 | 0.245 | 0.0546 | 0.937 |
| Collinsella | 0.002 | 0.00151 | 0.121 | 0.0771 | 0.937 |
| Cloacibacillus | 6.61e-05 | 7.52e-07 | 1.58 | 0.0843 | 0.937 |
| unclassified_Enterobacteriaceae | 0.00272 | 0.00958 | -0.547 | 0.0929 | 0.937 |
| unclassified_Veillonellaceae | 0.0034 | 0.000438 | 0.889 | 0.105 | 0.937 |
| Odoribacter | 0.0107 | 0.00399 | 0.429 | 0.108 | 0.937 |
| 1-68 | 1.89e-05 | 1.48e-05 | 0.0985 | 0.111 | 0.937 |
| Enterococcus | 0.000126 | 0.000116 | 0.038 | 0.118 | 0.937 |
| Adlercreutzia | 4.99e-05 | 0.000124 | -0.39 | 0.134 | 0.937 |
| Slackia | 0.000142 | 5.68e-05 | 0.392 | 0.152 | 0.937 |
| Prevotella | 0.167 | 0.216 | -0.111 | 0.159 | 0.937 |
| SMB53 | 0.0013 | 0.00146 | -0.0515 | 0.168 | 0.937 |
| unclassified_Planococcaceae | 2.56e-05 | 1.5e-05 | 0.22 | 0.169 | 0.937 |

**Figure S2.** Relative abundance of selected bacterial families at 12 months in the PD and LFD groups.

Boxplots showing the distribution of log10-transformed relative abundances for selected bacterial families in the personalized diet (PD) and low-FODMAP diet (LFD) groups at the 12-month follow-up. Relative abundances were log10-transformed after adding a small pseudocount (1e−6) to avoid undefined values for zero counts. Boxes represent the interquartile range (IQR), with the median indicated by the central line, and whiskers extending to 1.5× IQR. Each box summarizes the distribution across participants within each dietary group. Families displayed were selected based on their relative differences between groups in the exploratory differential abundance analysis. Longitudinal trajectories for selected genera

To relate cross-sectional differences observed at 12 months to temporal microbiome dynamics, we visualized longitudinal trajectories for selected genera commonly implicated in IBS and dietary fiber fermentation (Pittayanon et al., Gastroenterology, 2019; DOI: 10.1053/j.gastro.2018.10.049; Louis & Flint, Environ Microbiol, 2017; DOI: 10.1111/1462-2920.13589). Values are presented as mean ± 95% confidence intervals across baseline, 6 weeks, 6 months, and 12 months.
Pittayanon R et al. Gut microbiota in patients with irritable bowel syndrome—A systematic review. Gastroenterology. 2019 https://doi.org/10.1053/j.gastro.2018.10.049

**Figure S3.** Longitudinal trajectories of selected bacterial genera across the study period.

Mean relative abundances (±95% confidence intervals) of selected bacterial genera in the personalized diet (PD) and low-FODMAP diet (LFD) groups at baseline, 6 weeks, 6 months, and 12 months. Genera shown include Faecalibacterium, Roseburia, Bifidobacterium, Prevotella, Bacteroides, and unclassified Lachnospiraceae, which are commonly associated with fiber fermentation, short-chain fatty acid production, and gut microbial stability. Points represent group means and vertical error bars represent 95% confidence intervals. These trajectories are presented to contextualize cross- sectional differences observed at 12 months within the broader longitudinal dynamics of microbiome composition.

**Supplementary Table S3.** PD vs LFD at 12 months (functional proxy scores). (PD n=39, LFD n=31)

| Proxy score | PD mean | LFD mean | Diff (PD-LFD) | p (MWU) | q (FDR) |
| --- | --- | --- | --- | --- | --- |
| Propionate | 0.4074 | 0.4327 | -0.02534 | 0.1934 | 0.8861 |
| Lactate_prod | 0.00493 | 0.003237 | 0.001693 | 0.2371 | 0.8861 |
| Butyrate | 0.1612 | 0.1521 | 0.009095 | 0.3323 | 0.8861 |
| Sulfate/H2S | 0.003127 | 0.002416 | 0.0007112 | 0.5345 | 0.9039 |
| Mucin | 0.2261 | 0.2091 | 0.01694 | 0.6532 | 0.9039 |
| Lactate_util | 0.01236 | 0.01266 | -0.0002978 | 0.7228 | 0.9039 |
| BSH | 0.1998 | 0.1798 | 0.02003 | 0.8315 | 0.9039 |
| Methane | 9.187e-05 | 7.162e-05 | 2.025e-05 | 0.9039 | 0.9039 |

No PD–LFD contrasts at 12 months reached FDR significance in this taxonomy-based proxy analysis. The largest (uncorrected) differences were modest in magnitude.

For readability we show the key fixed effects (Time terms in PD reference group, and Group×Time interactions).

**Butyrate proxy**

| term | Coef. | Std.Err. | z | P>\|z\| |
| --- | --- | --- | --- | --- |
| group[T.lfd | 0.198 | 0.157 | 1.265 | 0.206 |
| Time 6w | 0.147 | 0.128 | 1.149 | 0.251 |
| Time 6m | 0.164 | 0.128 | 1.282 | 0.200 |
| Time 12m | 0.337 | 0.128 | 2.640 | 0.008 |
| group[T.lfd:Time 6w | -0.070 | 0.192 | -0.364 | 0.716 |
| group[T.lfd:Time 6m | -0.174 | 0.192 | -0.905 | 0.365 |
| group[T.lfd:Time 12m | -0.251 | 0.192 | -1.311 | 0.190 |

**BSH proxy**

| term | Coef. | Std.Err. | z | P>\|z\| |
| --- | --- | --- | --- | --- |
| group[T.lfd | -0.070 | 0.246 | -0.285 | 0.776 |
| Time 6w | -0.040 | 0.128 | -0.312 | 0.755 |
| Time 6m | 0.170 | 0.128 | 1.336 | 0.182 |
| Time 12m | -0.107 | 0.128 | -0.839 | 0.402 |
| group[T.lfd:Time 6w | 0.059 | 0.192 | 0.307 | 0.759 |
| group[T.lfd:Time 6m | -0.417 | 0.192 | -2.174 | 0.030 |
| group[T.lfd:Time 12m | 0.029 | 0.192 | 0.152 | 0.880 |

**Sulfate/H2S proxy**

| term | Coef. | Std.Err. | z | P>\|z\| |
| --- | --- | --- | --- | --- |
| group[T.lfd | -0.771 | 0.586 | -1.316 | 0.188 |
| Time 6w | 0.168 | 0.357 | 0.471 | 0.638 |
| Time 6m | -0.207 | 0.357 | -0.579 | 0.563 |
| Time 12m | -0.691 | 0.357 | -1.934 | 0.053 |
| group[T.lfd:Time 6w | 0.082 | 0.537 | 0.153 | 0.878 |
| group[T.lfd:Time 6m | 0.256 | 0.537 | 0.476 | 0.634 |
| group[T.lfd:Time 12m | 0.872 | 0.537 | 1.625 | 0.104 |

**Mucin proxy**

| term | Coef. | Std.Err. | z | P>\|z\| |
| --- | --- | --- | --- | --- |
| group[T.lfd | -0.105 | 0.228 | -0.460 | 0.646 |
| Time 6w | 0.004 | 0.124 | 0.031 | 0.975 |
| Time 6m | 0.272 | 0.124 | 2.190 | 0.029 |
| Time 12m | 0.086 | 0.124 | 0.690 | 0.490 |
| group[T.lfd:Time 6w | 0.029 | 0.187 | 0.157 | 0.875 |
| group[T.lfd:Time 6m | -0.343 | 0.187 | -1.839 | 0.066 |
| group[T.lfd:Time 12m | 0.000 | 0.187 | 0.000 | 1.000 |

**Notes:** Proxy scores represent taxonomy-based functional proxies calculated as the summed relative abundance of taxa associated with each metabolic function. Differences between PD and LFD groups at 12 months were tested using the Mann–Whitney U (MWU) test. Reported z values correspond to the standardized test statistic derived from the Mann–Whitney U test. P values were adjusted for multiple comparisons using the Benjamini–Hochberg false discovery rate (FDR) procedure and are reported as q (FDR) values. In the longitudinal mixed-effects models, Coef. indicates the estimated fixed-effect coefficient, Std. Err. the standard error, z the Wald test statistic, and P>|z| the associated two-sided p value.

**Figure S4.** Longitudinal changes in butyrate production potential in the PD and LFD groups.

Estimated butyrate production potential across the study period in the personalized diet (PD) and low-FODMAP diet (LFD) groups at baseline, 6 weeks, 6 months, and 12 months. The butyrate proxy score represents the summed relative abundance of bacterial taxa commonly associated with butyrate production. Points represent group means and vertical error bars indicate 95% confidence intervals. These proxy scores provide an approximate indication of functional shifts in microbiome metabolic potential over time.

**Figure S5.** Longitudinal changes in bile salt hydrolase (BSH) deconjugation potential in the PD and LFD groups.

Estimated bile salt hydrolase (BSH) deconjugation potential across the study period in the personalized diet (PD) and low-FODMAP diet (LFD) groups at baseline, 6 weeks, 6 months, and 12 months. The BSH proxy score represents the summed relative abundance of bacterial taxa commonly associated with bile salt hydrolase activity. Points represent group means and vertical error bars indicate 95% confidence intervals. These proxy scores provide an approximate indication of functional shifts in microbiome bile acid metabolism over time.

**Figure S6.** Longitudinal changes in sulfate reduction and hydrogen sulfide (H₂S) production potential in the PD and LFD groups.

Estimated sulfate reduction and hydrogen sulfide (H₂S) production potential across the study period in the personalized diet (PD) and low-FODMAP diet (LFD) groups at baseline, 6 weeks, 6 months, and 12 months. The sulfate/H₂S proxy score represents the summed relative abundance of bacterial taxa associated with sulfate-reducing metabolism and potential hydrogen sulfide production. Points represent group means and vertical error bars indicate 95% confidence intervals. These proxy scores provide an approximate indication of longitudinal changes in microbial sulfur metabolism.

**Figure S7.** Longitudinal changes in lactate production potential in the PD and LFD groups.

Estimated lactate production potential across the study period in the personalized diet (PD) and low-FODMAP diet (LFD) groups at baseline, 6 weeks, 6 months, and 12 months. The lactate-production proxy score represents the summed relative abundance of bacterial taxa associated with lactate-producing metabolism. Points represent group means and vertical error bars indicate 95% confidence intervals. These proxy scores provide an approximate indication of longitudinal shifts in microbial lactate production capacity during the follow-up period.

**Supplementary Table S4. Follow-up lifestyle factors, medication use, and health-perception indicators, by group and visit.**

| **Item** | **PD 6-mo (n=58)** | **LFD 6-mo (n=41)** | **p** | **PD 12-mo (n=40)** | **LFD 12-mo (n=29)** | **p** |
| --- | --- | --- | --- | --- | --- | --- |
| **Disease status change** |  |  | 0.250 |  |  | 0.020* |
| Improved | 31 (53.4) | 15 (36.6) |  | 23 (57.5) | 7 (24.1) |  |
| Unchanged | 20 (34.5) | 18 (43.9) |  | 9 (22.5) | 12 (41.4) |  |
| Not improved | 7 (12.1) | 8 (19.5) |  | 8 (20.0) | 10 (34.5) |  |
| Health problem (flu/COVID-like), Yes | 25 (43.1) | 12 (29.3) | 0.207 | 18 (45.0) | 10 (34.5) | 0.460 |
| COVID-19 infection, Yes | 1 (1.7) | 0 (0.0) | 1.000 | 3 (7.5) | 0 (0.0) | 0.258 |
| International travel, Yes | 9 (15.5) | 3 (7.3) | 0.349 | 7 (17.5) | 4 (13.8) | 0.750 |
| Surgical operation, Yes† | 8 (13.8) | 1 (2.4) | 0.076 | 3 (7.5) | 1 (3.4) | 0.634 |
| Antibiotic use, Yes | 25 (43.1) | 13 (31.7) | 0.297 | 14 (35.0) | 9 (31.0) | 0.800 |
| Antibiotic duration, days (mean ± SD) | 13.0 ± 13.2 | 11.0 ± 8.5 | 0.576 | 9.5 ± 3.9 | 13.0 ± 10.2 | 0.349 |
| Hospitalized > 2 days, Yes | 7 (12.1) | 1 (2.4) | 0.135 | 4 (10.0) | 2 (6.9) | 1.000 |
| Colonoscopy, Yes | 2 (3.4) | 1 (2.4) | 1.000 | 2 (5.0) | 0 (0.0) | 0.506 |
| Probiotic use, Yes | 8 (13.8) | 7 (17.1) | 0.778 | 8 (20.0) | 5 (17.2) | 1.000 |
| Prebiotic use, Yes | 8 (13.8) | 8 (19.5) | 0.581 | 5 (12.5) | 1 (3.4) | 0.389 |
| **Dietary adherence** |  |  | 0.118 |  |  | 0.010* |
| Always (76–100%) | 4 (6.9) | 0 (0.0) |  | 2 (5.0) | 0 (0.0) |  |
| Frequently (51–75%) | 14 (24.1) | 13 (31.7) |  | 8 (20.0) | 9 (31.0) |  |
| Sometimes (25–50%) | 29 (50.0) | 16 (39.0) |  | 20 (50.0) | 5 (17.2) |  |
| Rarely (1–25%) | 11 (19.0) | 10 (24.4) |  | 10 (25.0) | 15 (51.7) |  |
| Never (0%) | 0 (0.0) | 2 (4.9) |  | 0 (0.0) | 0 (0.0) |  |

*Data are n (%) unless otherwise stated. Between-group p-values used Fisher’s exact test for binary items, an exact conditional test with fixed margins and the Pearson χ² statistic for multi-category items (all feasible tables enumerated), and Welch’s t test for antibiotic duration. *p < 0.05. †The 12-month surgical-operation entry has been corrected from a transposed Yes/No coding in the previous version. These follow-up exposure and adherence comparisons are exploratory and not adjusted for multiplicity. Denominators reflect participants assessed at each visit (PD 6-month n=58; LFD 6-month n=41; PD 12-month n=40; LFD 12-month n=29). PD, microbiome-guided personalized diet; LFD, low-FODMAP diet.*

**Supplementary Table S5.** Between-group comparison of self-reported antibiotic exposure during the 12-month follow-up period in the personalized diet (PD) and low-FODMAP diet (LFD) groups.

| **Variable** | **PD group** | **LFD group** | **Between-group difference / effect size** | **Test** | **p value** |
| --- | --- | --- | --- | --- | --- |
| Any antibiotic use, 6-month visit (n exposed/N total) | 25/58 (43.1%) | 13/41 (31.7%) | ARD +11.4% (95% CI –8.0 to +29.0) | Fisher’s exact | 0.297 |
| Any antibiotic use, 12-month visit (n exposed/N total) | 14/40 (35.0%) | 9/29 (31.0%) | ARD +4.0% (95% CI –18.3 to +24.7) | Fisher’s exact | 0.800 |
| Duration of antibiotic use among exposed participants, 6 months (days, mean ± SD) | 13.0 ± 13.2 (n = 25) | 11.0 ± 8.5 (n = 13) | +2.0 days | Welch’s t | 0.576 |
| Duration of antibiotic use among exposed participants, 12 months (days, mean ± SD) | 9.5 ± 3.9 (n = 14) | 13.0 ± 10.2 (n = 9) | −3.5 days | Welch’s t | 0.349 |

*Abbreviations: PD, personalized diet; LFD, low-FODMAP diet; ARD, absolute risk difference; CI, confidence interval; SD, standard deviation. CIs are Newcombe hybrid-score intervals for the absolute risk difference; the Fisher exact test was used for the significance test.*

**Supplementary Table S6. Analytic sample table**

| **Analysis set** | **Baseline** | **6 weeks** | **6 months** | **12 months** |
| --- | --- | --- | --- | --- |
| Clinical outcomes | PD 70 / LFD 51 | PD 70 / LFD 51 | PD 58 / LFD 41 | PD 40 / LFD 29 |
| Available microbiome profiles (cross-sectional) | PD 70 / LFD 51† | PD 70 / LFD 51† | PD 56 / LFD 40 | PD 39 / LFD 31 |
| Four-time-point microbiome complete cases | PD 39 / LFD 31 | PD 39 / LFD 31 | PD 39 / LFD 31 | PD 39 / LFD 31 |
| Clinical + microbiome linked set | Not used | Not used | Not used | PD 39 / LFD 29 |
| Beta-diversity PERMANOVA | PD 70 / LFD 51† | PD 70 / LFD 51† | PD 56 / LFD 40 | PD 39 / LFD 31 |
| PERMDISP | Not reported | PD 39 / LFD 31‡ | PD 56 / LFD 40 | PD 39 / LFD 31 |

*Values are numbers of participants contributing data (PD / LFD). Questionnaire completion and stool-specimen availability were domain-specific. †Baseline and 6-week cross-sectional/PERMANOVA counts refer to the 6-week-completer cohort reported for the parent trial. ‡The available 6-week PERMDISP used the four-time-point complete-case subset and is a sensitivity analysis; it did not use the full 6-week PERMANOVA sample. At 6 and 12 months, PERMANOVA and PERMDISP used identical samples and Bray–Curtis matrices. PD, personalized diet; LFD, low-FODMAP diet.*

# Supplementary Table S7a. Primary PERMDISP analysis using the same Bray–Curtis matrix and samples as the corresponding PERMANOVA

| **Time point** | **PD n** | **LFD n** | **PERMANOVA pseudo-F** | **R²** | **p** | **F (df)** | **Permutation p** |
| --- | --- | --- | --- | --- | --- | --- | --- |
| 6 months | 56 | 40 | 3.411 | 0.035 | 0.011 | 0.121 (1,94) | 0.733 |
| 12 months | 39 | 31 | 1.494 | 0.0215 | 0.160 | 1.0 (1,68) | 0.319 |

# Supplementary Table S7b. Taxonomic-level sensitivity analysis

| **Taxonomic level** | **Time point** | **PERMDISP F (df)** | **Permutation p** | **Conclusion** |
| --- | --- | --- | --- | --- |
| Species | 6 months | 0.121(1,94) | 0.733 | No evidence of unequal dispersion |
| Species | 12 months | 1.0 (1,68) | 0.319 | No evidence of unequal dispersion |
| Genus | 6 months | 1.109 (1,94) | 0.291 | No evidence of unequal dispersion |
| Genus | 12 months | 1.026 (1,68) | 0.319 | No evidence of unequal dispersion |
| Phylum | 6 months | 0.170 (1,94) | 0.692 | No evidence of unequal dispersion |
| Phylum | 12 months | 0.011 (1,68) | 0.922 | No evidence of unequal dispersion |

**Methods and interpretation.** Bray–Curtis dissimilarities were calculated from the recovered relative-abundance profiles. Distances to the spatial median of each diet group were calculated in principal-coordinate space with the small-sample bias adjustment, and group dispersions were compared using 9,999 residual permutations. At 6 and 12 months, PERMDISP used the same participants and Bray–Curtis matrices as the corresponding PERMANOVA. Neither time point showed a statistically significant PD–LFD dispersion difference.

**Six-week sensitivity analysis.** PERMDISP was non-significant in the four-time-point complete-case microbiome subset (PD n=39; LFD n=31; F(1,68) = 1.83; p = 0.178). The corresponding 6-week PERMANOVA used the larger cross-sectional sample. This sensitivity result cannot establish that the full-sample PERMANOVA finding was independent of dispersion; the nominal full-sample result is treated as exploratory and is not used to infer centroid separation.

**Supplementary Table S8. Twelve-month responder proportions by baseline IBS severity.**

| Baseline severity | PD responders, n/N (%) | LFD responders, n/N (%) |
| --- | --- | --- |
| Mild | 1/2 (50.0) | 1/3 (33.3) |
| Moderate | 14/16 (87.5) | 6/14 (42.9) |
| Severe | 10/22 (45.5) | 3/12 (25.0) |

*Responder = reduction of at least 50 points in IBS-SSS from baseline. Cells are small, particularly the mild stratum; proportions are descriptive and were not formally tested. Subgroup responder totals sum to the overall 12-month responder counts (PD 25/40; LFD 10/29).*

# CONSORT 2010 Checklist

*Manuscript: Long-term Microbiome and Clinical Effects of a Microbiome-Guided Personalized Diet versus Low-FODMAP Diet in Irritable Bowel Syndrome: A 12-Month Follow-Up Randomized Controlled Trial (MS 250328137).*

This report is a 12-month follow-up of a registered, multicenter randomized controlled trial (ClinicalTrials.gov NCT05646186). Items relating to the original randomization, allocation concealment, and implementation are reported in the parent trial (reference 12) and summarized in the Methods. Page numbers refer to the manuscript file (Main_text); tables and figures are submitted as separate files and are cited at the indicated manuscript pages.

| **Section/Topic** | **Item** | **Checklist item** | **Reported (section / page)** |
| --- | --- | --- | --- |
| **Title and abstract** | 1a | Identification as a randomized trial in the title | Title page (p. 1); title states "…Randomized Controlled Trial" |
|  | 1b | Structured summary of trial design, methods, results, and conclusions | Abstract (pp. 1–2; single-paragraph/unstructured per journal policy) |
| **Introduction** | 2a | Scientific background and explanation of rationale | Introduction (pp. 2–3) |
|  | 2b | Specific objectives or hypotheses | Introduction, final paragraph (p. 3); Abstract (p. 2) |
| **Methods** | 3a | Description of trial design (e.g., parallel, factorial), including allocation ratio | Methods 2.1 (p. 3): parallel, open-label, PROBE design; 1:1 allocation in parent trial |
|  | 3b | Important changes to methods after commencement, with reasons | Methods 2.2 (p. 4); Discussion/Limitations (p. 14): pre-planned 12-month follow-up; long-term analyses specified for this phase |
|  | 4a | Eligibility criteria for participants | Methods 2.1 (p. 3) and 2.2.1 (pp. 4–5) |
|  | 4b | Settings and locations where data were collected | Methods 2.1 (p. 3): four gastroenterology centres, Türkiye |
|  | 5 | Interventions for each group with sufficient detail to allow replication | Methods 2.1.2 and Follow-Up Protocol (pp. 4–5) |
|  | 6a | Completely defined pre-specified primary and secondary outcome measures | Methods 2.2.2 (p. 5) and 2.3–2.3.4 (pp. 5–6) |
|  | 6b | Any changes to trial outcomes after the trial commenced, with reasons | Methods 2.5 (p. 6); Discussion/Limitations (p. 14) |
|  | 7a | How sample size was determined | Methods (p. 7) and Discussion/Limitations (p. 14): parent trial powered for the 6-week primary endpoint; follow-up not prospectively powered for long-term contrasts |
|  | 7b | Explanation of any interim analyses and stopping guidelines | Not applicable (no interim analyses or stopping rules for the follow-up) |
|  | 8a | Method used to generate the random allocation sequence | Methods 2.1 (p. 3); as reported in the parent trial (ref 12) |
|  | 8b | Type of randomization; details of any restriction | Methods 2.1 (p. 3); parent trial (ref 12) |
|  | 9 | Mechanism used to implement the allocation sequence (concealment) | Methods 2.1 (p. 3); parent trial (ref 12) |
|  | 10 | Who generated the allocation sequence, enrolled participants, and assigned them | Methods 2.1 (p. 3); parent trial (ref 12) |
|  | 11a | Blinding — if done, who was blinded after assignment and how | Methods 2.1 (p. 3): open-label intervention with blinded outcome assessment (PROBE) |
|  | 11b | If relevant, description of the similarity of interventions | Not applicable (open-label dietary interventions) |
|  | 12a | Statistical methods used to compare groups for primary and secondary outcomes | Methods 2.5 (p. 6): linear mixed-effects models; Fisher exact test with Newcombe intervals; PERMANOVA; PERMDISP |
|  | 12b | Methods for additional analyses, such as subgroup and adjusted analyses | Methods 2.5 (pp. 6–7): baseline-severity subgroups; multiple-imputation sensitivity; functional proxies |
| **Results** | 13a | Numbers randomly assigned, receiving intended treatment, and analyzed for the primary outcome | Figure 1 (CONSORT flow diagram; submitted separately); Results 3.1 (pp. 8–9) |
|  | 13b | Losses and exclusions after randomization, together with reasons | Figure 1 (CONSORT); Results 3.1 (p. 8); Table 5 (attrition; cited p. 11) |
|  | 14a | Dates defining the periods of recruitment and follow-up | Methods 2.1 (p. 3): recruitment August 2022–May 2023; followed to 12 months |
|  | 14b | Why the trial ended or was stopped | Not applicable (follow-up completed as planned) |
|  | 15 | A table showing baseline demographic and clinical characteristics for each group | Table 1 (submitted separately; cited p. 9); baseline described in Results 3.1 (p. 9) |
|  | 16 | For each group, number of participants included in each analysis | Figure 1 (CONSORT); Supplementary Table S6; Results 3.1 (pp. 8–9) |
|  | 17a | For each outcome, results for each group and the estimated effect size and its precision | Results 3.2–3.3 (pp. 9–11); Tables 2–4 (submitted separately; cited pp. 9–10); Figures 2–3 |
|  | 17b | For binary outcomes, presentation of both absolute and relative effect sizes | Table 3 (12-month responder absolute risk difference with Newcombe 95% CI; cited p. 9) |
|  | 18 | Results of any other analyses performed, including subgroup and adjusted analyses | Results 3.3–3.5 (pp. 10–12); Supplementary tables/figures (labeled exploratory) |
|  | 19 | All important harms or unintended effects in each group | Results 3.5 (p. 12) and Supplementary Table S4 (follow-up medication/health exposures). |
| **Discussion** | 20 | Trial limitations, addressing sources of potential bias and imprecision | Discussion / Limitations (pp. 14–15) |
|  | 21 | Generalizability (external validity, applicability) of the trial findings | Discussion (pp. 13–15) |
|  | 22 | Interpretation consistent with results, balancing benefits and harms | Discussion and Conclusion (pp. 12–15) |
| **Other information** | 23 | Registration number and name of trial registry | Methods 2.1 (p. 3) and Ethics approval (p. 16): ClinicalTrials.gov NCT05646186 |
|  | 24 | Where the full trial protocol can be accessed, if available | Methods / Data availability statement (pp. 15–16). |
|  | 25 | Sources of funding and other support; role of funders | Funding statement (p. 16) |

*CONSORT 2010 checklist adapted for a follow-up report of a registered randomized controlled trial. "Not applicable" denotes items not relevant to this observational long-term follow-up. Page numbers correspond to the manuscript file (Main_text, 19-page rendering)*
